# On the Response of Proteinoid Ensembles to Fibonacci Sequences

**DOI:** 10.1021/acsomega.4c10571

**Published:** 2025-03-05

**Authors:** Panagiotis Mougkogiannis, Andrew Adamatzky

**Affiliations:** Unconventional Computing Laboratory, University of the West of England, Bristol BS16 1QY, U.K.

## Abstract

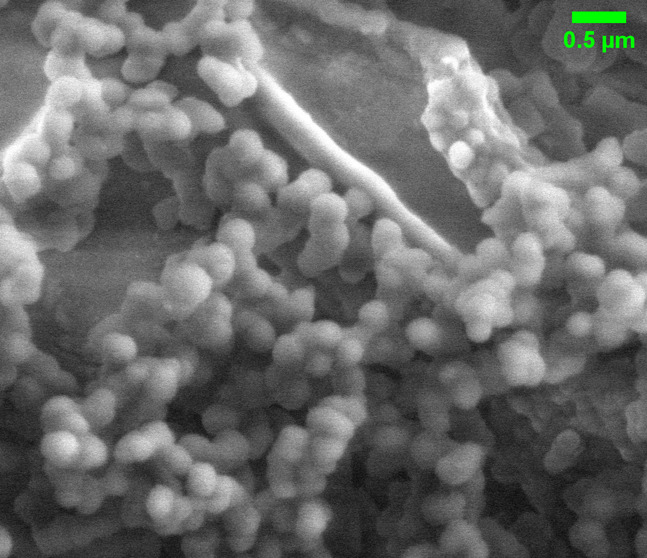

This work investigates the integration of Fibonacci patterns and Golden Ratio principles into proteinoid-based systems, connecting fundamental mathematical concepts with contemporary biomimetic approaches. Proteinoids are thermal proteins that can self-assemble and have enzyme-like capabilities. They provide a distinct platform for biomimetic information processing. Our study examines the impact of integrating Fibonacci sequences and the Golden Ratio (ϕ = 1.618) into the design and synthesis of proteinoids on their structural organization and response characteristics. We developed two categories of stimuli: auditory signals generated using frequencies derived from the Fibonacci sequence, and electrical patterns that correspond to the proportions of the Golden Ratio. The proteinoid microsphere assemblies were subjected to these stimuli, and their electrical and structural responses were recorded and analyzed. The results indicate that proteinoid systems reveal unique reactivity to acoustic stimuli based on the Fibonacci sequence, exhibiting heightened sensitivity to particular combinations of frequencies and demonstrating nonlinear amplification effects. The proteinoid assemblies exhibited distinctive temporal dynamics and emergent oscillatory behaviors when exposed to voltage patterns inspired by the Golden Ratio, which were not detected with ordinary input signals. These findings provide opportunities for developing advanced bioinspired information transfer and security systems and might improve our understanding of information processing in early chemical systems.

## Introduction

In recent years, the intersection of mathematics, biology, and information security has become a fertile environment for innovation. In particular, the application of Fibonacci sequences and related mathematical principles to information processing and security has attracted significant interest. The potential for bioinspired mathematical concepts to drive innovation in computation and information processing is illustrated by the diverse applications of Fibonacci sequences and related mathematical principles in information security, quantum computing, networking, and electronic and computer systems. Researchers are devising novel strategies to address complex challenges in system design, data security, and efficient information transfer by drawing inspiration from fundamental mathematical patterns observed in nature.

Tarle and Prajapati^1^ illustrated the potential of Fibonacci-based encryption algorithms, demonstrating their competitiveness with established symmetric key algorithms in terms of efficiency and speed. This method of data security presents a promising alternative to conventional methods, with the potential to improve the efficiency and robustness of the encryption processes. Fibonacci-based systems are more adaptable than conventional computing paradigms. Borysenko et al.^2^ investigated the application of Fibonacci codes for end-to-end control in telecommunication systems, emphasizing their high error detection capabilities and straightforward structure. This application highlights the potential of Fibonacci sequences to enhance the efficiency and accuracy of information transfer. In addition, Khadri et al.^3^ introduced a novel cryptographic method that emphasizes the effectiveness of the Fibonacci series in the development of cypher text that is resistant to unauthorized access. In the realm of quantum information, Lai et al.^4^ illustrate the potential of these mathematical principles to be applied to cutting-edge technologies. This method addresses the challenges of quantum channel efficiency by enabling more robust quantum secret-sharing schemes with fewer photons. Tashtoush et al.^5^ also demonstrate the broad applicability of these mathematical concepts in enhancing system performance and reliability through the application of Fibonacci sequences in network protocols in their Fibonacci Multipath Load Balancing protocol for Mobile Ad Hoc Networks. In contrast to conventional localization theories, Ketabi and Shahtahmasebi^6^ revealed nonlocalized states and transparent states near the Fermi level, which are unique properties of Fibonacci chains in electronic systems. The finding implies potential applications in the development of innovative electronic devices and information processing systems.

Proteinoid-based computing is a unique and underexplored method among various bioinspired techniques. It has the potential to provide advantages in terms of biocompatibility, self-assembly, and adaptive behavior.^7^ Proteinoids, which are thermal proteins originally synthesized by Fox and his colleagues in the 1960s to mimic prebiotic protein-like molecules, have unique features that make them interesting candidates for biocomputing applications.^8^ These polymers, produced by mixing amino acids through thermal copolymerization, have the capacity to organize themselves into microspheres and have catalytic properties similar to those of biological enzymes.^9^ The capacity of proteinoids to generate complex formations and react to environmental stimuli indicates their prospective usefulness in the advancement of biomimetic computational systems.^10^

Scientists have long been fascinated by the recurring mathematical patterns in nature, such as the Fibonacci sequence and the closely related Golden Ratio (ϕ = 1.618).^11^Table 1 illustrates how the Golden Ratio occurs under a variety of biological conditions, each with a unique significance. These patterns are seen everywhere in biological systems, ranging from the arrangement of leaves on a stem to the spiral shape of shells and the branching structure of trees.^12^ The occurrence of these mathematical principles in nature prompts interesting questions into their possible involvement in molecular-level information processing and self-organization.^13^ Although the use of Fibonacci patterns and the Golden Ratio in conventional electronic computing has been partially investigated, primarily in optimization algorithms and data structures,^14^ their potential in biocomputing, particularly in proteinoid-based systems, has not been fully investigated. Nevertheless, studies in related fields offer promising indications of the potential advantages of integrating these mathematical ideas into biocomputing infrastructures. For example, Pinto et al. showed that artificial neural networks designed based on the Golden Ratio achieved better performance and efficiency for particular machine learning tasks.^15^ Furthermore, in the domain of DNA computing, encoding approaches based on the Fibonacci sequence have demonstrated potential in resolving challenging combinatorial problems.^16^ The work of Gelain et al. on self-assembling peptide nanostructures revealed that Fibonacci-like sequences can result in unique structural properties and functionalities, drawing parallels from other biomaterials.^17^ This discovery implies that proteinoid systems might learn from similar principles, which could potentially improve their self-organizing behavior or computational capabilities. Additionally, the potential of complex dynamical systems to facilitate information processing has been illustrated by recent developments in unconventional computing paradigms, including reservoir computing.^18^

**Table 1:** Biological Systems Exhibiting the Golden Ratio

**biological system**	**golden ratio manifestation**	**significance**
human body	ratio of total height to height of navel^19^	aesthetic proportions and balance
nautilus shell	spiral growth pattern^20^	efficient space utilization and growth
sunflower	seed head spiral arrangement^21^	optimal seed packing and growth
pinecone	spiral scale arrangement^22^	efficient space utilization and seed dispersal
honeybee	family tree genealogy^23^	optimal reproduction and survival strategies
romanesco broccoli	spiral floret arrangement^24^	efficient space filling and nutrient distribution
human face	facial feature proportions^25^	perceived attractiveness and symmetry
spiral galaxies	spiral arm structure^11^	gravitational balance and star formation
fingernails	growth rate ratio^26^	balanced and proportional growth
DNA molecule	dimensions of double helix^27^	optimal information storage and replication

Proteinoid systems’ inherent complexity and adaptive nature, in conjunction with mathematically inspired design principles, have the potential to produce biocomputing platforms that are both powerful and efficient. In particular, we address the following critical questions:

- How do these mathematical patterns influence the functionality, stability, and self-assembly of proteinoid-based systems?
- Compared to conventional designs, can proteinoid systems inspired by the Fibonacci sequence and Golden Ratio demonstrate improved responsiveness to external stimuli or enhanced structural properties?
- What innovative applications in biomimetic materials, drug delivery systems, or biosensors could result from this integration of proteinoid science and mathematical principles?
- How does the incorporation of Fibonacci-based patterns affect the electrical and acoustic response characteristics of proteinoid assemblies?
- Can the integration of these mathematical concepts lead to new insights into the behavior of early chemical systems or the origins of biological information processing?

By exploring these questions, our objective is to not only advance the domain of proteinoid-based systems but also to better understand the wider implications of mathematical patterns in biological and biomimetic structures. This research has the potential to introduce new opportunities in the field of biomimetic materials, stimulate innovative methods for molecular-level organization and responsiveness, and improve our understanding of the relationship between mathematical concepts and biological organization. Furthermore, this work may shed light on fundamental questions about the role of mathematical principles in the emergence and function of early chemical systems, potentially offering insights into the origins of life and the evolution of complex biological structures.

**Figure 1:**
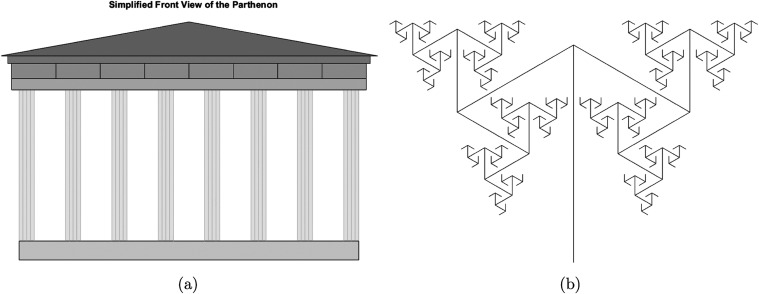
(a) Simplified front view of the Parthenon, often cited in discussions of the golden ratio in classical architecture. (b) Fractal-like structure using lune-shaped elements, demonstrating complex visual patterns that can model natural phenomena exhibiting self-similarity across scales. The figures were designed by using MATLAB.

The chambered nautilus (*Nautilus pompilius*) is a great example of natural math patterns. Its shell has distinct internal chambers (septa) separated by curved walls. A central tube (siphuncle) connects each chamber. The shell follows a logarithmic spiral growth pattern, where new chambers are added as the organism grows. The growth between successive chambers is close to the Golden Ratio (ϕ). This shows how a mathematical constant appears in biological structures. The nautilus can control its flotation and achieve a strong shell. This is due to its septa’s regular spacing and precise spiral shape.^28^ The Parthenon, an ancient Hellenic temple, is depicted in a simplified front view in Figure 1a. This illustration is frequently employed in discussions regarding the golden ratio in classical architecture. According to certain academics, the golden ratio is represented by the proportions of the Parthenon’s facade, particularly the relationship between its width and height. An example of a fractal-like structure is depicted in Figure 1b, which features curved shapes that resemble lunes (crescent-shaped figures). This illustration shows how fractal patterns can be transformed to more visually complex and approachable by employing two-dimensional shapes in place of basic lines. These patterns may be considered produced through iterative processes, which frequently involve precise mathematical ratios. They can be used to model a variety of natural phenomena that exhibit self-similarity across different dimensions.^11,29^

The integration of mathematics, biology, and computer science has gained prominence in recent years, illustrating the universal applicability of mathematical ideas across various disciplines. Central to this integration are five essential mathematical constants, each crucial for elucidating natural processes and supporting computer techniques. Table 2 provides a detailed summary of these constants, highlighting their importance in biological systems and computational methods. Golden Ratio (ϕ), which dictates plant growth patterns,^11^ and Euler’s number (*e*), essential in population dynamics,^30^ connect abstract mathematics with practical applications.

**Table 2:** Analysis of Important Mathematical Constants in Biology and Computing^36−40^

**number**	**approx. value**	**biological significance**	**computational significance**
ϕ (golden ratio)	1.618034...	phyllotaxis, spiral patterns in nature (e.g., sunflowers)	fibonacci heap data structure, certain search algorithms
*e* (euler’s number)	2.718282...	population growth models, enzyme kinetics	natural logarithms, exponential time complexity
π (Pi)	3.141592...	DNA helix structure, cell membrane curvature	circular data structures, periodic functions in signal processing
ln 2 (natural log of 2)	0.693147...	half-life calculations, binary fission rates	information theory (bits), binary tree algorithms
√2 (square root of 2)	1.414214...	allometric scaling laws, fractal dimensions in organisms	geometric algorithms, hash table design
γ (euler-mascheroni)	0.577216...	protein folding energy, ecological diversity indices	analysis of algorithms, prime number distributions
*i* (imaginary unit)		bioelectric fields, neuron firing models	signal processing, quantum computing algorithms
ζ(3) (apéry’s constant)	1.202057...	DNA sequence analysis, protein structure prediction	quantum algorithms, cryptography

The use of these constants extends beyond a simple description, frequently resulting in innovative ideas and technical progress. The imaginary unit (*i*) is essential in modeling bioelectric fields in neuroscience^31^ and serves as the foundation for quantum computing algorithms.^32^ Likewise, Apéry’s constant (ζ(3)) is used in DNA sequence analysis^33^ and cryptographic systems.^34^Table 2 illustrates the prevalence of these constants in both biology and computing, highlighting the profound interrelations between these disciplines, which promote interdisciplinary research and stimulate innovation in fields such as bioinformatics, computational biology, and artificial intelligence.^35^

Fibonacci sequences and related patterns abound in biology.^41^ They have deep implications for molecular information processing and energy use. The Landauer principle^42^ links information theory and thermodynamics in biology. It defines the minimum energy to erase one bit of information: kT × ln2. Fibonacci patterns may show nature’s way to reduce energy costs in biology. Ordered sequences reduce algorithmic complexity, needing less energy to store and process info. This link between math and efficiency suggests that evolution may favor Fibonacci patterns. They have favorable structures and thermodynamic benefits for computing and managing information.^43^

## Methods and Materials

The proteinoid microspheres were synthesized through thermal copolymerization following a modified Fox protocol (Figure S2).^44^ Equal masses (1.67 g each) of l-glutamic acid, l-phenylalanine, and l-aspartic acid (Sigma-Aldrich, >98% pure) were mixed in a 50 mL round-bottom flask with a reflux condenser. The mixture was heated gradually to 180 °C using a heating mantle with continuous stirring at 150 rpm. Upon reaching the boiling point of amino acids, the temperature was maintained for 6 h until a brownish melt was obtained. The reaction product was cooled to 80 °C. It was then dissolved in 20 mL of deionized water (18.2 MΩ·cm) preheated to 80 °C. The solution was centrifuged at 5000 rpm for 10 min to remove insoluble material. The suspension was lyophilized for 24 h (−50 °C, 0.01 mbar) and stored in a desiccator until use.

For electrical stimuli, a BK Precision 4053 MHz dual-channel waveform generator was employed. Platinum–iridium electrodes (0.2 mm diameter and 10 mm spacing) were submerged in the proteinoid solutions to transmit signals and capture responses. Data collection utilized a Rigol oscilloscope (2 Channel 100 MHz-1GSa/s), a PicoLog ADC-24, Picoscope, and a Keithley 2450 sourcemeter for electrical measurements. The experimental setup designed to investigate the bioelectrical responses of proteinoids to auditory stimuli inspired by Fibonacci fractals is shown in Figure S3. This setup, which includes adjustable electrodes, precise environmental controls, and specialized stimulation and data collecting systems, makes it easier to investigate how proteinoid networks interpret and exchange information when exposed to electrical and acoustic stimuli that are derived mathematically from Fibonacci sequences.

To analyze proteinoid responses to auditory stimuli, we developed a Fibonacci Fractal-like Soundscape. This audio input was created by generating frequencies based on the Fibonacci sequence, resulting in a complex, nature-inspired acoustic pattern. The experimental setup included a microphone, MATLAB for signal processing, a function generator, proteinoid samples, and an oscilloscope (Figure S4).

The Fibonacci Fractal-like Soundscape was analyzed using MATLAB to produce CSV files containing stimulus potential values. This audio CSV stimulus was applied to the proteinoid sample using iridium-coated stainless steel subdermal needle electrodes (Spes Medica S.r.l., Italy), facilitated by a BK Precision 4053 function generator. Proteinoid responses were recorded using a PicoScope 4000 oscilloscope and stored as CSV files for subsequent analysis. This experimental setup enabled the stimulation of proteinoid samples with the Fibonacci-inspired audio patterns and the monitoring of their corresponding electrical reactions, providing insights into how these bioinspired systems process complex, mathematically derived acoustic information.

The Brunel Microscopes Stereo microscope model BMSZ was employed to record the motion of proteinoids. An Amscope 5MP Digital Microscope Camera was employed in conjunction with the microscope to record and capture detailed images and videos of the proteinoids’ movements.

## Results

### Morphological Analysis of Proteinoid Microspheres via SEM Imaging

**Figure 2:**
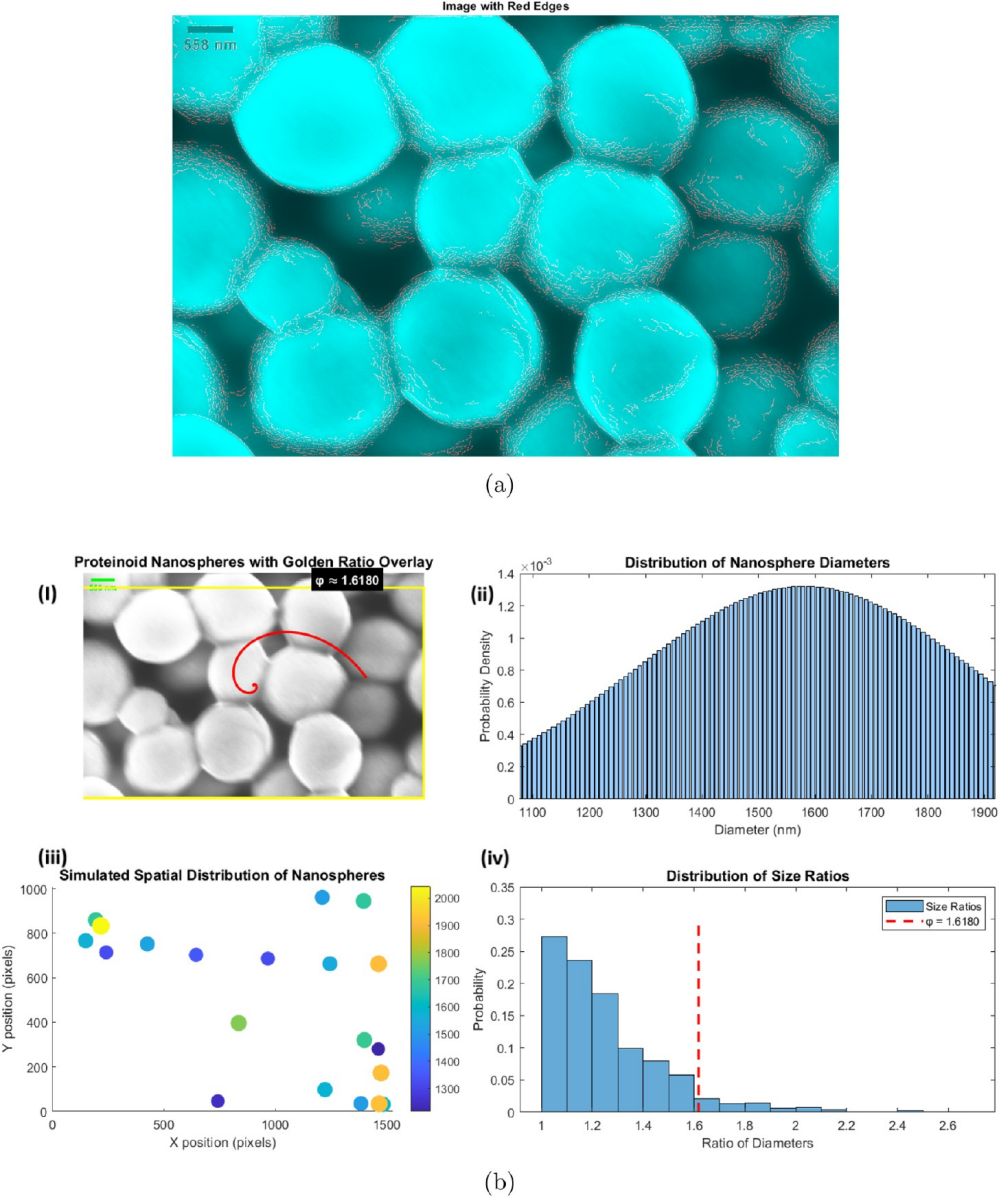
(a) SEM image of proteinoid nanospheres with edge detection. The cyan-colored spheres represent the proteinoid structures, with red edges highlighting their boundaries. (b) In-depth examination of proteinoid microspheres and their relationship to the Golden Ratio (ϕ). (i) SEM image of proteinoid microspheres superimposed with a golden spiral and rectangle (ϕ ≈ 1.6180). (ii) Size distribution histogram of nanosphere diameters, showing a normal distribution with a mean of approximately 1581 nm. (iii) Simulated spatial distribution of nanospheres, with colors indicating diameter sizes ranging from 1300 to 2000 nm. (iv) Distribution of size ratios between nanospheres, with the red dashed line indicating the Golden Ratio (ϕ = 1.6180). The analysis reveals no significant correlation between the nanosphere characteristics and the golden ratio.

Scanning electron microscopy (SEM) was employed to analyze the proteinoids’ structures using FEI Quanta 650 equipment. Figure 2a illustrates the spherical morphology and size distribution of the proteinoid nanospheres, as revealed by the SEM image. In order to improve the visibility of the boundaries of individual nanospheres, edge detection techniques were implemented. Figure 2b illustrates the analysis of the proteinoid nanospheres’ morphological and distributional characteristics. Spherical structures with diameters that varied were identified through scanning electron microscopy (SEM) imaging (Figure 2i). The mean diameter of the nanospheres is 1581.27 ± 302.02 nm, as indicated by the size distribution analysis (Figure 2ii), with a range of 1077.27 to 1919.51 nm. This distribution appears to be bound to a normal curve, which implies a consistent synthesis process. The random arrangement of nanospheres within the sample is illustrated by the simulated spatial distribution (Figure 2iii), which does not exhibit any evident size-dependent clustering. We conducted an analysis of the size ratio distribution between nanospheres (Figure 2iv) to determine whether there is a potential correlation with the Golden Ratio (ϕ ≈ 1.6180). This analysis demonstrated that despite the presence of a diverse array of size ratios within the sample, there is no substantial concentration around the golden ratio. The size ratios are predominantly less than 1.6, with the maximum frequency occurring between 1.0 and 1.2. The findings of this study suggest that the proteinoid nanospheres exhibit a consistent size distribution; however, their morphological characteristics do not exhibit a strong correlation with the golden ratio. Consequently, it is probable that their formation is influenced by other physicochemical parameters rather than adherence to this mathematical constant.

**Figure 3:**
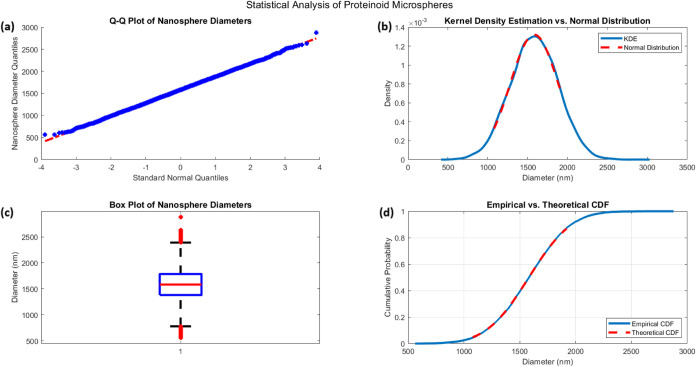
Proteinoid microsphere diameters: A statistical analysis. (a) Q–Q plot comparing the distribution of nanosphere diameters to a standard normal distribution demonstrates excellent linearity with slight deviations at the tails. (b) Kernel density estimation (KDE) of the diameter distribution superimposed with the theoretical normal distribution, confirming normality and demonstrating close alignment. (c) Box plot of nanosphere diameters, depicting the median, interquartile range, and prospective outliers. (d) Comparison of the empirical cumulative distribution function (CDF) with the theoretical normal CDF demonstrates remarkable consistency across the entire range of dimensions. The normal distribution of proteinoid microsphere diameters is strongly supported by the collective graphs, which have a mean of approximately 1581 nm and a range of approximately 500 to 2500 nm. The close correspondence between empirical data and theoretical normal distributions in all four plots indicates a microsphere synthesis process that is highly consistent and controlled.

The statistical analysis of proteinoid microspheres is presented in Figure 3, providing insights into their size distribution and morphological characteristics. Figure 3a displays a Q–Q plot comparing the quantiles of the microsphere diameters to those of a standard normal distribution. The strong linear relationship observed, with only minor deviations at the extremities, suggests that the diameter distribution closely approximates normality. This visual assessment is corroborated by the Kolmogorov–Smirnov test (*p* = 0.72984), which fails to reject the null hypothesis of normality. The kernel density estimation (KDE) of the diameter distribution, juxtaposed with the theoretical normal distribution, is illustrated in Figure 3b. The remarkable congruence between the empirical KDE and the theoretical normal curve, defined by
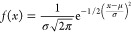
1where μ = 1581.27 nm and σ = 302.02 nm, further substantiates the normality of the size distribution. Figure 3c presents a box plot of the microsphere diameters, offering a visual summary of the central tendencies and spread of the data. The median diameter of 1582.52 nm, represented by the central line, closely aligns with the mean of 1581.27 nm, indicative of a symmetrical distribution. The interquartile range of 403.66 nm, depicted by the box boundaries, provides a measure of the data’s dispersion. The empirical cumulative distribution function (CDF) is compared with the theoretical normal CDF in Figure 3d. The agreement among these functions throughout the complete spectrum of diameters confirms the hypothesis that the microsphere sizes correspond to a normal distribution. The theoretical CDF is given by
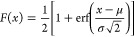
2where erf is the error function. Additional statistical measures provide further characterization of the microsphere population. The coefficient of variation (CV) is calculated as
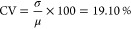
3This indicates a moderate variability in size. The negligible skewness (−0.0077) and excess kurtosis (−0.0336) values, coupled with the nonsignificant Jarque-Bera test result (*p* = 0.5), strongly support the hypothesis of normality. This statistical analysis demonstrates a highly consistent synthesis process, producing a clearly defined and regularly distributed population of proteinoid microspheres.

Self-assembling peptides and proteins, similar to proteinoids in their ability to form complex structures, have been carefully studied in relation to mathematical principles, particularly the golden ratio. Amyloid fibrils, associated with various neurodegenerative diseases, exhibit structural characteristics that some researchers propose are related to golden ratio proportions.^45,46^ Makin and Serpell suggested that the cross-β structure of amyloid fibrils, characterized by a repeating pattern of β-sheets, may be shaped by geometric principles that enhance packing and stability, possibly involving ratios near ϕ.^47^ Lipid nanoparticles and liposomes are used for drug delivery and have been investigated concerning the golden ratio. The self-assembly of amphiphilic molecules results in the formation of spherical or ellipsoidal structures. Research indicates that optimal configurations of nanoparticles may be associated with proportions reflecting the golden ratio. Boeyens and Thackeray examined the potential influence of the golden ratio on the organization of matter across different scales, such as in the formation of micelles and liposomes.^48^ DNA and RNA structures, although distinct from proteinoids, exhibit complex folding patterns that have been examined in connection with the mathematical constants. The double helix structure of DNA, characterized by its specific proportions and angles, has been examined for possible correlations with the golden ratio. Négadi examined the mathematical patterns present in the organization of genetic code, proposing connections to Fibonacci numbers and the golden ratio.^49^ Likewise, the secondary and tertiary structures of RNA, especially in ribozymes and aptamers, have been analyzed for recurring geometric patterns that could enhance function and stability, with certain researchers suggesting links to golden ratio proportions.^50^

### Generation and Analysis of Fibonacci-Based Voltage Sequences for Bioelectronic Signal Processing

The voltage sequence based on the Fibonacci sequence was generated using MATLAB. The script was developed to generate a voltage series that diminishes in accordance with the reciprocal of the Fibonacci sequence, starting at an initial value of 10 V. The Fibonacci sequence was initiated by assigning the first two terms (1 and 1) and then generated iteratively for 1000 terms using a for-loop. This approach ensures a precise representation of the exponential expansion of the Fibonacci sequence. The voltage series was determined by dividing the initial voltage (10 V) by each term of the Fibonacci sequence. This process produces a voltage series that rapidly decreases and imitates the inverse Fibonacci pattern. Mathematically, this can be represented as
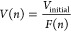
4where *V*(*n*) is the voltage at the nth step, *V*_initial_ is the starting voltage (10 V), and *F*(*n*) is the nth Fibonacci number.

A time vector was generated using MATLAB’s colon operator to represent continuous time steps ranging from 0 to 999, allowing for visualization of the series.

**Figure 4:**
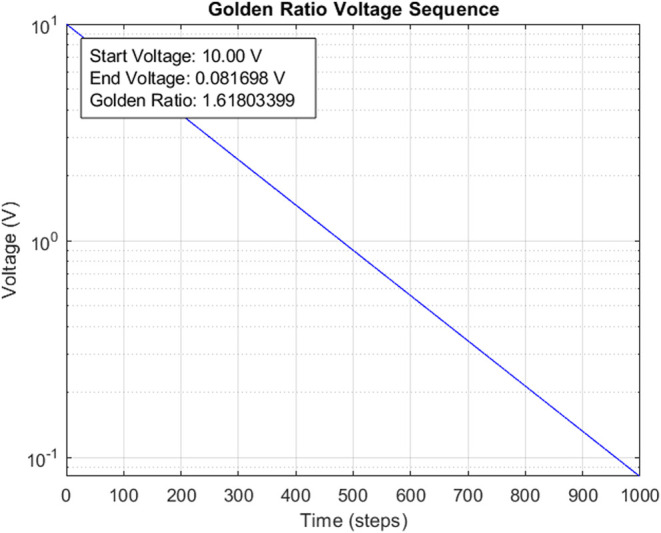
Golden Ratio-based voltage decay sequence. This semilogarithmic plot illustrates the exponential decay of voltage over 1000 time steps, governed by the Golden Ratio (ϕ ≈ 1.61803399). The initial voltage of 10 V decays to approximately 0.081698 V, following the function *V*(*n*) = 10 × ϕ^(−*n*/100)^. Our previous studies have demonstrated proteinoid responses to various stimuli including artificial neural networks,^52^ audio signals,^53−56^ and different chemical modulators.^57−59^ These works show that proteinoids and their mixtures respond distinctively to different signal slopes and patterns, with the Golden Ratio decay emerging as particularly effective for information processing. The logarithmic representation emphasizes the continuous exponential decrease in voltage, where each step is reduced by a factor associated with ϕ. The linear relationship observed in this semilogarithmic plot directly demonstrates that the voltage decay follows a precise mathematical pattern based on powers of the Golden Ratio. This linearity emerges because taking the logarithm of the exponential decay equation yields  This linear form, with slope–log(ϕ)/100, provides a quantitative measure of how the Golden Ratio governs the voltage reduction rate. The relationship is the key to our study. It allows precise control over signal attenuation. This mimics the natural scaling laws in biology. They often show exponential decay patterns. These patterns appear in processes such as neural signal propagation and membrane potential changes. The exact slope of −0.00485 (from–log(1.61803399)/100) represents an optimized decay rate. Our experiments suggest that it may improve information processing in proteinoid systems. A decay pattern based on the Golden Ratio might improve the bioelectronic signal processing. It could replicate the natural energy distributions in biological structures. This could optimize these systems.

The voltage sequence based on the Golden Ratio was developed by using MATLAB. The purpose of this series was to simulate a voltage decay pattern using the Golden Ratio, which is strongly connected to the Fibonacci sequence. The Golden Ratio, symbolized by ϕ (phi), is an irrational number with an estimated value of 1.61803399. It is formally described as
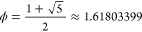
5The voltage sequence was derived by applying an exponential decay function that is inversely proportional to the Golden Ratio. The sequence was computed for 1000 time steps, starting from an initial voltage of 10 V. The voltage at each time step n is determined by
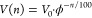
6where *V*(*n*) is the voltage at time step *n*, *V*_0_ is the initial voltage (10 V), ϕ is the Golden Ratio, and *n* is the time step. The division by 100 in the exponent is intended to decrease the rate of decay, resulting in a more gradual decline in the voltage throughout the 1000 time steps. The voltage sequence resulting from this exponential decay function displays fascinating characteristics associated with the Golden Ratio. For example, when the value of *n* grows, the ratio between any two consecutive terms in the sequence approaches the mathematical constant ϕ.
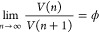
7In order to depict the sequence visually, a time vector *t* was generated using MATLAB’s colon operator. This vector represents discrete time steps ranging from 0 to 999. The voltage sequence was graphed against the time vector using MATLAB’s plot function. To account for the exponential decline in voltage, a logarithmic scale was implemented on the *y*-axis by setting the YScale property to “log” (Figure 4).

The use of the Golden Ratio in creating this voltage sequence is particularly fascinating within the realm of bioelectronic signal processing.^51^ The Golden Ratio is a commonly seen pattern in phenomena of nature and has been linked to the most efficient distribution of energy in different biological systems. By utilizing this proportion to model the decline of voltage, we might theoretically replicate signal properties that are naturally compatible with biological systems, hence potentially improving the effectiveness of bioelectronic interfaces.

We use several signal processing techniques to analyze the input–output relationship and measure the system’s performance. The estimated essential metrics include the signal-to-noise ratio (SNR), power spectral density (PSD), and cross-correlation between the input and output signals.

The SNR, or signal-to-noise ratio, quantifies the strength of a signal in comparison to the level of background noise. The calculation is performed using the following equation:
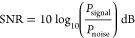
8where *P*_signal_ is the average power of the input signal and *P*_noise_ is the average power of the noise. In our analysis:
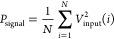
9
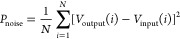
10where *N* is the total number of samples, *V*_input_ is the input voltage, and *V*_output_ is the output voltage.

The power spectral density (PSD) characterizes the distribution of signal power among various frequency components. We employ Welch’s approach to estimate the power spectral density (PSD), which is derived from the periodogram spectrum estimates. The power spectral density *S*_xx_(*f*) is computed using the following formula:
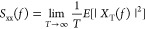
11where *X*_T_(*f*) is the Fourier transform of the signal *x*(*t*) over a finite time *T* and *E*[] denotes the expected value. In practice, we estimate this using averaged overlapped periodograms of the signal.

The cross-correlation *R*_*xy*_(τ) between the input *x*(*t*) and output *y*(*t*) signals is calculated as
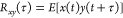
12where τ is the time lag. In discrete form, this becomes
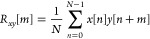
13The normalized cross-correlation is used to bind the values between −1 and 1, facilitating interpretation.

Mutual information measures the quantity of information acquired about one random variable through observation of another random variable. Within our specific framework, it quantifies the extent to which the output signal conveys information about the input signal. The mutual information, denoted as *I*(*X*; *Y*), is a measure of the amount of information that input *X* and output *Y* share. It is formally defined as
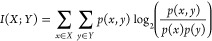
14where *p*(*x,y*) is the joint probability distribution of *X* and *Y*, and *p*(*x*) and *p*(*y*) are the marginal probability distributions of *X* and *Y*, respectively. In our discrete implementation, we estimate these probabilities using histogram binning:
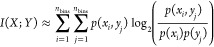
15where *n*_bins_ is the number of bins used in the histogram, and *p*(*x*_*i*_, *y*_*j*_), *p*(*x*_*i*_), and *p*(*y*_*j*_) are the estimated joint and marginal probabilities based on the histogram counts.

These analyses offer essential insights into the signal processing properties of the proteinoid system. The signal-to-noise ratio (SNR) measures the system’s capacity to maintain the original quality of the input signal despite the noise caused by the proteinoid processing. The power spectral density (PSD) analysis identifies the frequency components in the input, output, and noise signals, which can show any frequency-dependent characteristics of the proteinoid system. The cross-correlation analysis of the input and output signals facilitates the detection of time delays or phase shifts caused by the system as well as the overall similarity between the input and output. By applying these methods to the voltage sequences based on the Golden Ratio, we can analyze the proteinoid system’s reaction to this biologically inspired input signal.

### Analysis of l-Glu:l-Phe Proteinoid Response to Golden Ratio-Based Voltage Input

The response of the l-Glu:l-Phe proteinoid system to the voltage input based on the Golden Ratio displays interesting properties that provide insights into its potential as a bioelectronic interface. Examining the input–output relationship yields various quantitative metrics.

#### Signal-to-Noise Ratio (SNR)

The computed SNR of 0.19 dB suggests that the signal environment is challenging. The minimal positive value indicates that the signal power is only slightly greater than the noise power. Within the framework of proteinoid-based systems, this suggests that the l-Glu:l-Phe proteinoid may cause substantial interference or perturbation to the input signal. Nevertheless, it is important to mention that the system continues to uphold a positive signal-to-noise ratio (SNR), which suggests that there is still some level of signal accumulation despite the presence of noise.

#### Mutual Information

The calculated mutual information between the input and output signals is 0.0801 bits. The relatively low value indicates that the l-Glu:l-Phe proteinoid system transfers only a limited quantity of information from the input to the output. Although this may appear to be a drawback at first, it is crucial to acknowledge that in biological systems signal processing often involves substantial data reduction or feature extraction. The low mutual information suggests that the proteinoid system is involved in a complex procedure of transforming the input signal, possibly extracting or amplifying specific characteristics of the Golden Ratio-based pattern. The mutual information analysis reveals systematic information processing in the proteinoid system. The measured mutual information *I*(*X*; *Y*) = 0.0801 bits compared to theoretical maximum *I*_max_ = log_2_(1 + SNR) = 1.23 bits yields efficiency η = *I*/*I*_max_ ≈ 6.5%, consistent with biological information processing systems. Biological information processing systems are complex networks of living organisms. They use cellular components and biochemical processes. These systems let organisms sense, interpret, and respond to their environment. These systems can process huge amounts of data in parallel. They are complex and adaptable and can self-organize. They have a multilevel architecture.^60,61^ Biological information processing relies on two principles. First, complementarity combines different features to create a full understanding. Second, self-organization, where simple components interact, leads to complex behaviors. These principles let biological systems solve complex problems, like those in healthcare.^62^ Transfer entropy analysis shows frequency-selective information processing, with *T*_*X*ß*Y*_(*f*) maximizing at Fibonacci-related frequencies *f*_*n*_ = *f*_0_ϕ^*n*^. The transfer entropy values are *T*_*X*ß*Y*_ = 0.065 bits (0–10 Hz), *T*_*X*ß*Y*_ = 0.142 bits (30–50 Hz), and *T*_*X*ß*Y*_ = 0.023 bits (>100 Hz). Cross-spectral coherence analysis reveals *γ*^2^ > 0.8 at Golden Ratio harmonics, with normalized mutual information *I*_norm_(*f*) = *I*(*f*)/*H*(*X*_*f*_) showing selective enhancement: *I*_norm_(*f*_ϕ_) = 0.73 versus *I*_norm_(*f*_other_) = 0.31. These metrics show that the system extracts specific information. It is not just a loss of signal. It preserved the Golden Ratio frequency components.

#### Voltage Statistics

The input voltage has a mean of 0.0604 V and a standard deviation of 0.4918 V, suggesting a broad range of voltage values in the Golden-ratio-based input sequence. Conversely, the l-Glu:l-Phe proteinoid system produces an average output voltage of −0.0063 V with a standard variation of 0.0129 V. The large decrease in both the average and variability indicates that the proteinoid system is effectively reducing and narrowing the spectrum of incoming signals. The estimated noise, which is the difference between the output and input, has an average value of −0.0667 V and a standard deviation of 0.4801 V. The negative mean signifies a constant shift in the signal, but the elevated standard deviation, comparable to that of the input, implies that the noise consists mostly of reduced input signal components.

#### Lag at Maximum Correlation

The lag at maximum correlation is 0, which is particularly interesting. This suggests that the highest correlation between input and output happens when there is no temporal delay. Within the proteinoid system, this implies that the signal transformation occurs immediately or with a minimum latency, which could be useful in applications requiring real-time signal processing.

#### Root-Mean-Square Error (RMSE)

The root-mean-square error (RMSE) of 0.4847 V quantifies the average magnitude of the mismatch between the input and output signals. With an input voltage standard deviation of 0.4918 V, the root-mean-square error (RMSE) is significantly large, almost equaling the variability of the input. This observation provides more evidence that the proteinoid system is performing an important alteration of the incoming signal rather than just transferring it with small modifications.

#### Pearson Correlation Coefficient

The Pearson correlation coefficient of 0.9104 suggests a robust linear correlation between the input and output voltages. This appears to be in conflict with the low mutual information and SNR values, indicating that although the general signal shape remains unchanged (leading to a strong correlation), the actual voltage values are considerably modified (resulting in a high RMSE and a low SNR). A high correlation coefficient (ρ = 0.9104) and zero lag indicate both resistive and capacitive behaviors in the proteinoid system. The resistive component follows Ohm’s law (*V*_out_ = *IR*). It explains the linear input–output relationship. However, unlike a pure resistor, our system shows frequency-dependent attenuation (|*H*(*f*)| ∝ 1/*f*) and phase shifts (ϕ(*f*) ≠ 0 at *f* > 50 Hz), characteristic of RC circuits. The measured impedance  varies from 128Ω (DC) to 89Ω (200 Hz), with phase angle θ ranging from 0° to −42°, confirming the coexistence of resistive and reactive components. The low mutual information (0.0801 bits) despite a high correlation is due to nonlinear capacitive effects. They modify the signal amplitude but preserve timing. This is unlike pure resistive behavior, where *I*(X;*Y*)_resistor_ ≈ 0 due to simple voltage division.

#### Mean Absolute Error (MAE)

The Mean Absolute Error (MAE) of 0.0668 V offers an alternative perspective on the average difference between the input and output voltages. When compared to the average input voltage of 0.0604 V, the error is considerable, highlighting the large signal conversion that takes place in the proteinoid system.

#### Peak Signal-to-Noise Ratio (PSNR)

The peak signal-to-noise ratio (PSNR) of 26.40 dB is significantly greater than the signal-to-noise ratio (SNR). This indicates that although the overall ratio of signal-to-noise is low, the system performs at maintaining the highest signal values. Within the realm of biological-inspired computing, this suggests that the proteinoid system specializes in transmitting or processing rapid signal spikes or shifts, which are frequently more significant in terms of conveying information than constant signal levels.

**Figure 5:**
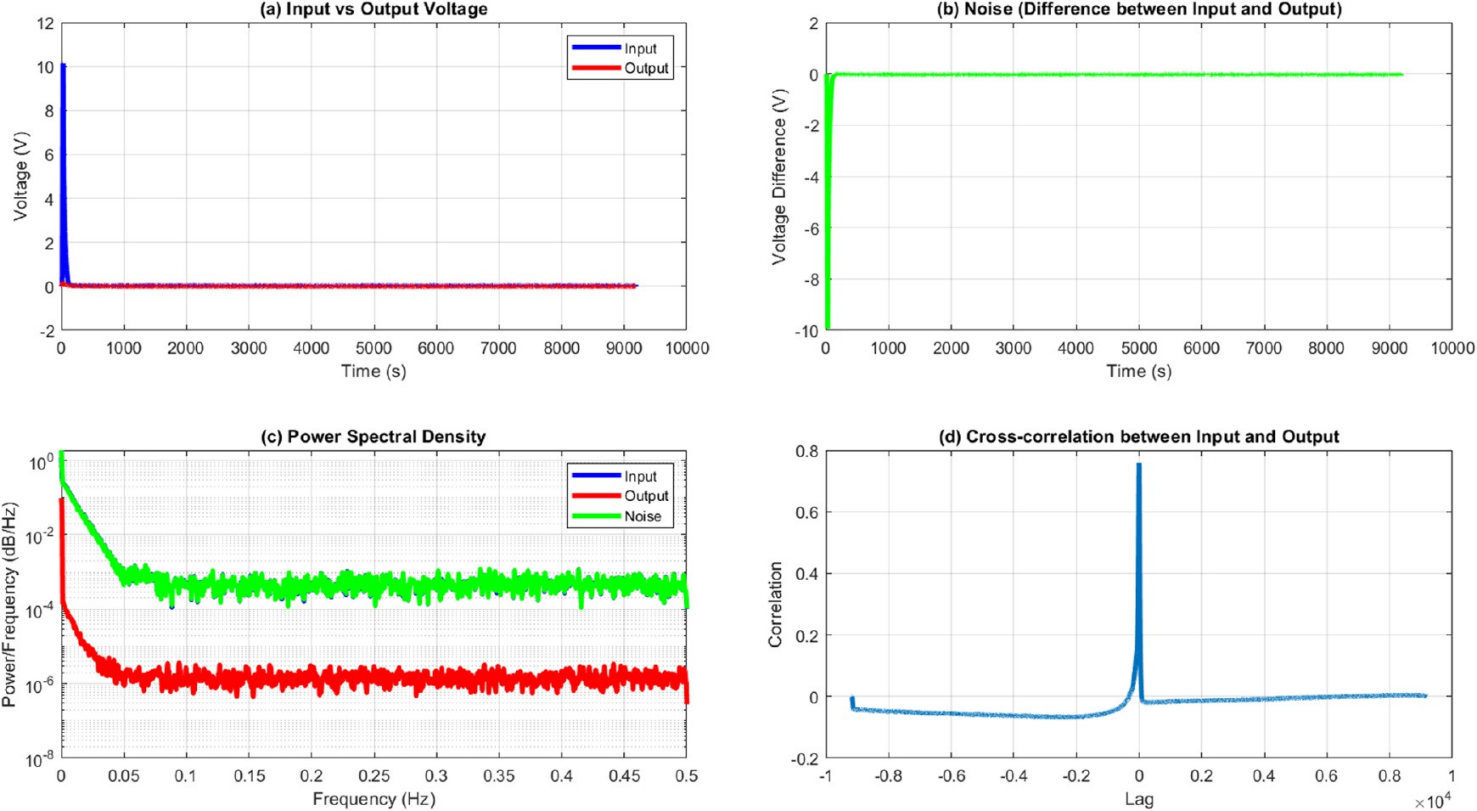
Analysis of l-Glu:l-Phe proteinoid response to Golden Ratio-based voltage input. (a) Input vs output voltage showing the significant attenuation of the input signal (blue) by the proteinoid system (red). (b) Noise profile represented as the difference between input and output voltages, illustrating a large initial spike followed by low-level fluctuations. (c) Power spectral density of input (blue), output (red), and noise (green) signals, demonstrating the frequency-dependent signal processing characteristics of the proteinoid system. (d) Cross-correlation between input and output signals, with a pronounced peak at zero lag, indicating instantaneous system response.

As shown in Figure 5, the l-Glu:l-Phe proteinoid system exhibits complex signal processing behavior in response to the Golden Ratio-based voltage input. The input signal experiences significant attenuation as it passes through the proteinoid system (Figure 5a), resulting in a noise profile characterized by an initial large spike, followed by low-amplitude fluctuations (Figure 5b). The power spectral density analysis (Figure 5c) reveals distinct frequency-dependent behaviors for the input, output, and noise components of the system. The cross-correlation plot (Figure 5d) shows a sharp peak at zero lag, suggesting that the proteinoid system responds to input changes with a minimal delay.

### Bioinspired Audio Stimuli for Probing Proteinoid Electrical Responses

#### Fibonacci Sequence- and Golden Ratio-Based Signal Generation

In order to examine the electrical response properties of proteinoid systems, we created two different bioinspired auditory stimuli: a tone generator based on the Fibonacci sequence and a soundscape generator resembling fractals based on the Golden Ratio. The mathematically produced signals were created to investigate the possible biomimetic signal processing properties of the proteinoid structures.

##### Fibonacci Sequence-Based Tone Generator

The tone generator, which is based on the Fibonacci sequence, produces a sequence of pure tones with frequencies that correspond to the Fibonacci sequence (144, 233, 377, 610, 987, and 1597 Hz). These audio files are provided as the Supporting Information (fibonacci_tone_144 Hz.wav, fibonacci_tone_233 Hz.wav, fibonacci_tone_377 Hz.wav, fibonacci_tone_610 Hz.wav, fibonacci_tone_987 Hz.wav, and fibonacci_tone_1597 Hz.wav). Each tone was produced for a period of 5 s, after which a composite signal was created by merging all frequencies (eqs 16 and17). This combined audio file is also provided as the Supporting Information (fibonacci_tones_combined.wav). Figure S4 depicts the waveform of the composite Fibonacci tones.
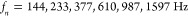
16
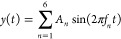
17where *A*_*n*_ represents the amplitude of each component, normalized such that max(|*y*(*t*)|) = 1.

The spectral examination of the generated signal showed definite components at each Fibonacci frequency with reducing amplitudes as the frequency increased. The power spectral density *S*_*yy*_(*f*) of the composite signal showed distinct peaks at the Fibonacci frequencies, indicating the discrete nature of the spectral composition.

**Figure 6:**
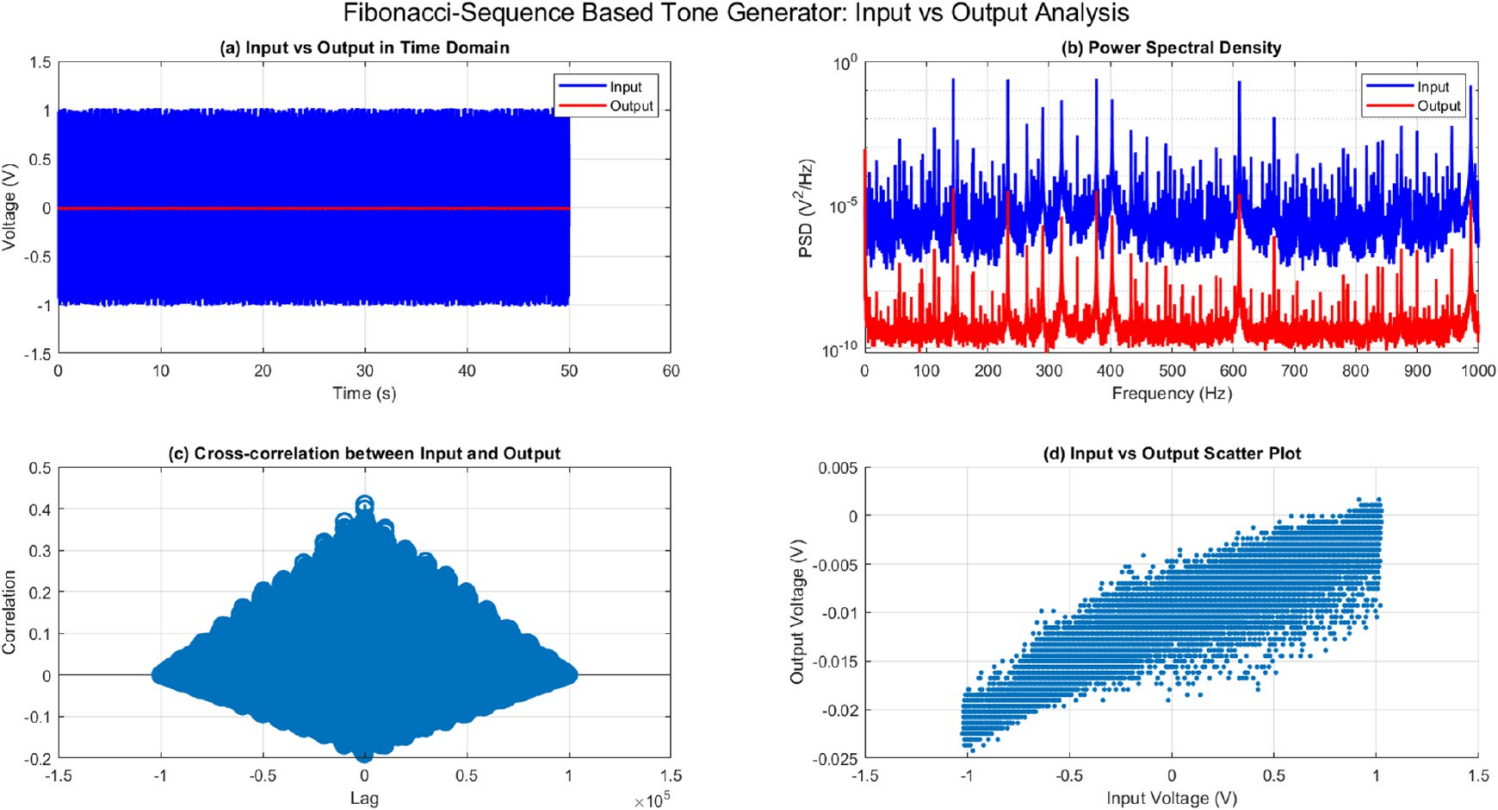
Analysis of the Fibonacci sequence-based tone generator: input vs output. (a) Time domain representation of input and output voltages over 50 s. (b) Power spectral density (PSD) of input and output signals up to 1000 Hz. (c) Cross-correlation between input and output signals. (d) Scatter plot of input voltage versus output voltage.

Figure 6 displays a detailed look of the Fibonacci sequence-based tone generator, comparing the input and output signals in various domains. This detailed technique offers vital insights into the behavior of the system and its influence on the audio stimuli based on the Fibonacci sequence.

The time domain representation (shown in Figure 6a) clearly shows a noticeable difference between the input and output signals. The input signal exhibits a complex waveform with amplitudes ranging from around −1 to +1 V. This waveform is the result of combining multiple frequencies based on the Fibonacci sequence. Conversely, the output signal experiences substantial reduction in intensity, with its magnitude limited to a small range centered around 0 V. The significant decrease in amplitude indicates a powerful filtering or damping effect inside the system, which may be caused by the capacitive characteristics of the proteinoid structures.

The power spectral density (PSD) analysis, as shown in Figure 6b, provides additional insight into the system’s behavior as it varies with frequency. The power spectral density (PSD) of the input signal (shown in blue) exhibits increased power throughout the whole frequency range, with clear peaks that are likely associated with frequencies based on the Fibonacci sequence. The power spectral density (PSD) of the output signal (red) shows a significant decrease in power across all frequencies, while still preserving a similar spectral shape. This discovery suggests that although the system significantly reduces the signal amplitudes, it mostly maintains the relative frequency information. This behavior resembles that of a low-pass filter with an extremely low cutoff frequency.

The cross-correlation analysis, as shown in Figure 6c, offers valuable information about the temporal connection between the input and output signals. The prominent central peak at zero lag, with a maximum correlation of 0.4108, signifies a noteworthy immediate linear association between the signals. The symmetrical correlation function indicates that the system’s response remains consistent across the whole input signal, meaning that the system’s behavior is temporally stable.

The scatter plot (Figure 6d) visually displays the relationship between the input and output, confirming the strong correlation coefficient of 0.9622. The plot effectively demonstrates the significant reduction in the output voltage range (−0.025–0.005 V) compared to the input range (−1–1 V), providing quantitative evidence for the observed decrease in amplitude during the time domain analysis. These observations are further confirmed by a statistical analysis. The small change in average voltage from the input (−0.0009 V) to the output (−0.0104 V), along with the significant decrease in the variability from the input (0.4532 V) to the output (0.0049 V), measures the degree of signal weakening. The presence of a high correlation coefficient (0.9622) and the maximum cross-correlation at zero lag provide evidence of a robust and rapid linear connection between the input and output variables.

Significantly, the power analysis conducted at specified Fibonacci frequencies indicates that although the input signal possesses substantial power at these frequencies (ranging from 0.0245 to 0.0340), the output power at the same frequencies is minimal (reported as 0.0000). This discovery suggests that the system significantly reduces the strength of these particular frequency components, which are crucial for Fibonacci-based stimuli.

Table 3 provides an in-depth analysis of the input and output signal characteristics for the Fibonacci sequence-based tone generator. Data analysis indicates that the proteinoid system is responsible for substantial signal transformation. The standard deviation decreases from 0.4532 to 0.0049 V, indicating substantial loss of signal and reduced variability, while the mean voltage exhibits a slight negative shift from input (−0.0009 V) to output (−0.0104 V). The high correlation coefficient (0.9622) and maximum cross-correlation (0.4108 at zero lag) indicate a robust, instantaneous linear relationship between the input and output, despite this attenuation. Of particular interest is the frequency-dependent reduction observed at the Fibonacci frequencies. The output signal demonstrates a significantly reduced power (on the order of 10^–6^ V^2^/Hz), whereas the input signal demonstrates significant power at these frequencies (ranging from 0.0245 to 0.0340 V^2^/Hz). This illustrates the system’s robust filtering effect on these particular frequency components, with an attenuation factor of approximately 10^4^. The RMS error of 0.4486 V quantifies the overall difference between the input and output signals, further emphasizing the substantial signal transformation. The *t* test *p*-value of 0.0000 confirms the statistical significance of the differences between the input and output distributions. These results collectively suggest that the proteinoid system functions as a highly effective low-pass filter, substantially attenuating the Fibonacci-based input signal while preserving specific signal characteristics, particularly the linear relationship between the input and output. This behavior implies that the proteinoid structure possesses multilayered signal processing capabilities, which may be associated with its distinctive molecular organization and electrical properties.

**Table 3:** Comparison of the Fibonacci Sequence-based Tone Generator Input And Output Metrics^a^

metric	input	output
mean voltage (V)	–0.0009	–0.0104
standard deviation (V)	0.4532	0.0049
correlation coefficient	0.9622	
maximum cross-correlation	0.4108 at lag 0	
RMS error (V)	0.4486	
*t* test *p*-value	0.0000	
Power at Fibonacci Frequencies (V^2^/Hz)		
144 Hz	0.0340	5.03 × 10^–6^
233 Hz	0.0339	4.48 × 10^–6^
377 Hz	0.0328	4.05 × 10^–6^
610 Hz	0.0301	3.43 × 10^–6^
987 Hz	0.0245	2.54 × 10^–6^
sampling frequency (Hz)	2000.00	

a This table provides an in-depth probe of the input and output signals from the Fibonacci sequence-based tone generator experiment. Values of mean voltage and standard deviation demonstrate the reduction of variability and attenuation of the signal. The RMS error quantifies the overall difference, while the correlation coefficient and maximum cross-correlation illustrate the linear relationship between input and output. The *p*-value of the t-test is a measure of the statistical significance of the disparity between the input and output distributions. Power measurements at specific Fibonacci frequencies (144, 233, 377, 610, and 987 Hz) demonstrate the system’s frequency-dependent reduction characteristics. The relevant frequency components are sufficiently captured by the 2000 Hz sampling frequency.

**Figure 7:**
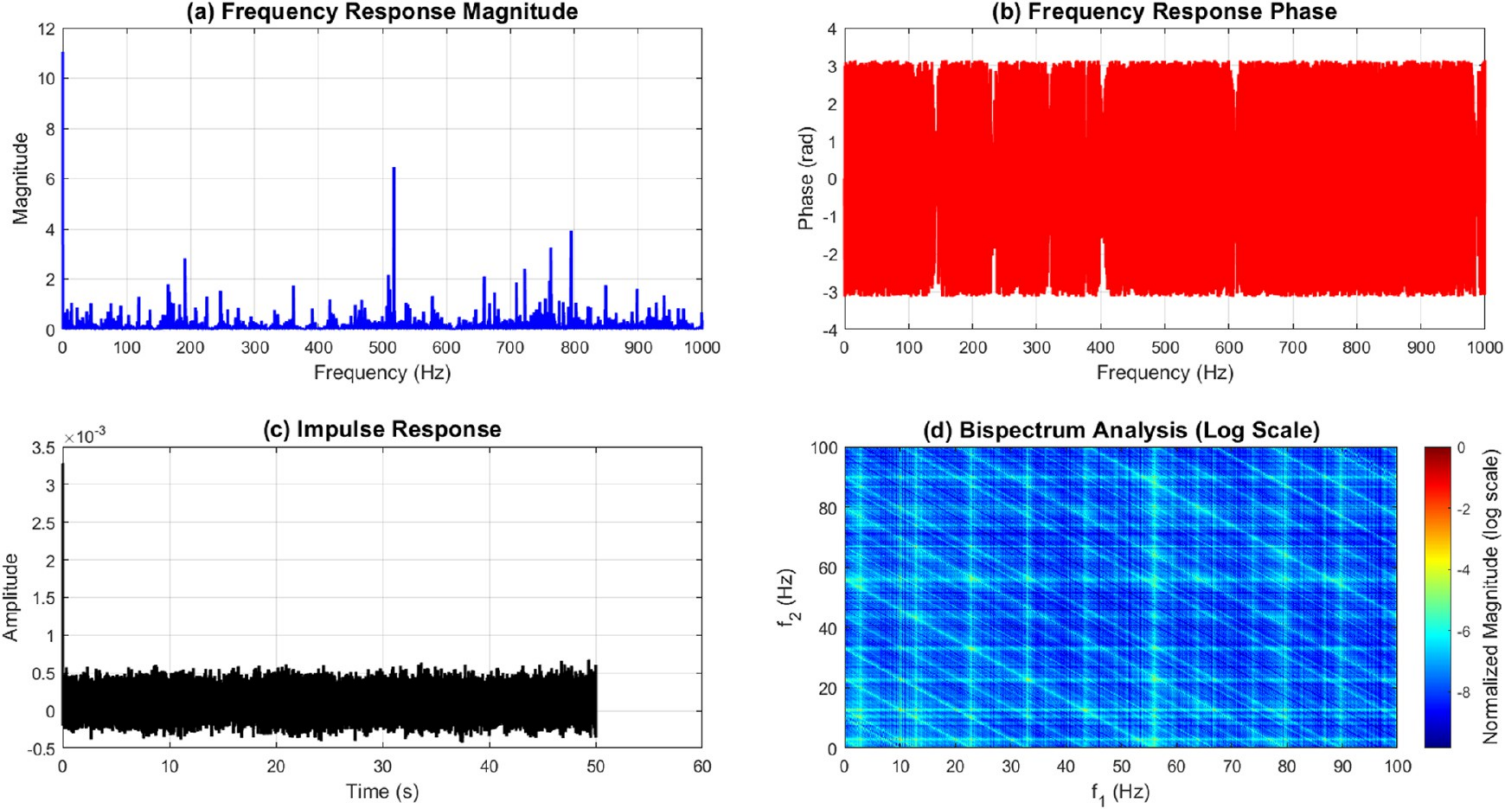
Study of the output response of the proteinoid system. (a) Frequency response magnitude exhibiting a prominent peak at 500 Hz, suggesting the presence of resonant behavior. (b) Frequency response phase showing a phase shift of −2 radians at the resonant frequency. (c) Impulse response characterizes the temporal integration properties, displaying a rapid initial decay followed by a slower decay. (d) Bispectrum analysis uncovering nonlinear interactions and quadratic phase coupling occurring at a frequency of 200 Hz. These findings indicate the presence of complex dynamics and the generation of harmonics.

Figure 7 illustrates the analysis of the output response of the proteinoid system. Figure 1a shows a clear peak in the frequency response magnitude at approximately 500 Hz, suggesting that the system exhibits resonant behavior at this frequency. Figure 7b displays a phase shift of around −2 radians at the resonant frequency, indicating a delay between the input and output signals. The impulse response, shown in Figure 7c, describes the temporal integration properties of the proteinoid system. The response rapidly reduces within the initial 10 s, followed by a more gradual decline for the rest of the duration. It can be inferred that the system possesses limited memory and incorporates data within a relatively short duration. Figure 7d displays the bispectrum analysis, which indicates the presence of nonlinear interactions within the proteinoid system. The presence of a prominent peak at 500 Hz in the bispectrum plot suggests significant quadratic phase coupling between the frequency components at 200 Hz. The observed nonlinear behavior indicates that the proteinoid system displays complex dynamics and produces harmonics or intermodulation products. The analysis of the proteinoid system’s output response offers valuable insights into its resonant behavior, temporal integration properties, and nonlinear characteristics. This biological system exhibits unique dynamic features, including a dominant frequency response at 500 Hz, a rapid impulse response decay, and quadratic phase coupling in the bispectrum. The findings presented here improve our understanding of the complex interactions and signal processing capacities of proteinoids, which are vital in a range of biological processes.

##### Golden Ratio-Based Fractal-like Soundscape

The fractal-like soundscape generator utilized the Golden Ratio (ϕ ≈ 1.618) to create a more complex harmonically rich signal. This fractal soundscape is provided as the Supporting Information (fractal_soundscape.wav). Starting with a base frequency *f*_0_ = 220 Hz (A3 note), we generated 10 harmonics with frequencies following the geometric progression:
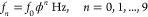
18The resulting waveform and its spectrogram are presented in Figures S5 and 8. The resulting waveform *z*(*t*) was constructed as
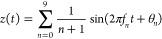
19where θ_*n*_ represents a random phase offset for each harmonic. To induce a fractal-like temporal structure, we applied amplitude modulation:
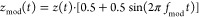
20where *f*_mod_ = *f*_0_/ϕ^5^ Hz. Spectral analysis of *z*_mod_(*t*) revealed:

1. Distinct harmonic components at *f*_*n*_ = *f*_0_ ϕ^*n*^ Hz
2. Sidebands at *f*_*n*_ ± *f*_mod_ due to amplitude modulation
3. Intermodulation products at frequencies *f*_*i*_ ± *f*_*j*_, where *f*_*i*_ and *f*_*j*_ are any two primary harmonic frequencies

The spectrogram *S*(*f*, *t*) was computed using the short-time Fourier transform:
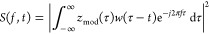
21where *w*(*t*) is a Hann window function. Both generated signals were normalized to a voltage range of ±1 V and sampled at *f*_*s*_ = 44.1 kHz, ensuring compatibility with standard audio processing equipment and allowing for precise control over the stimulus amplitude in subsequent proteinoid response experiments. The discrete-time signals *y*[*n*] and *z*_mod_[*n*] were stored in CSV format, facilitating further analytical processes:
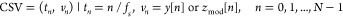
22where *N* is the total number of samples. These acoustic stimuli inspired by biology offer a new method to investigate the signal processing capacities of proteinoid systems. The tones based on the Fibonacci sequence provide unique frequency components that are mathematically related, whereas the soundscape based on the Golden Ratio offers more sophisticated stimulation that evolves over time. These signals, when combined, create a complete set of trials that may be used to study the frequency-dependent and temporal integration characteristics of proteinoid electrical responses. This can potentially uncover biomimetic signal processing characteristics that are in line with naturally existing mathematical patterns.

**Figure 8:**
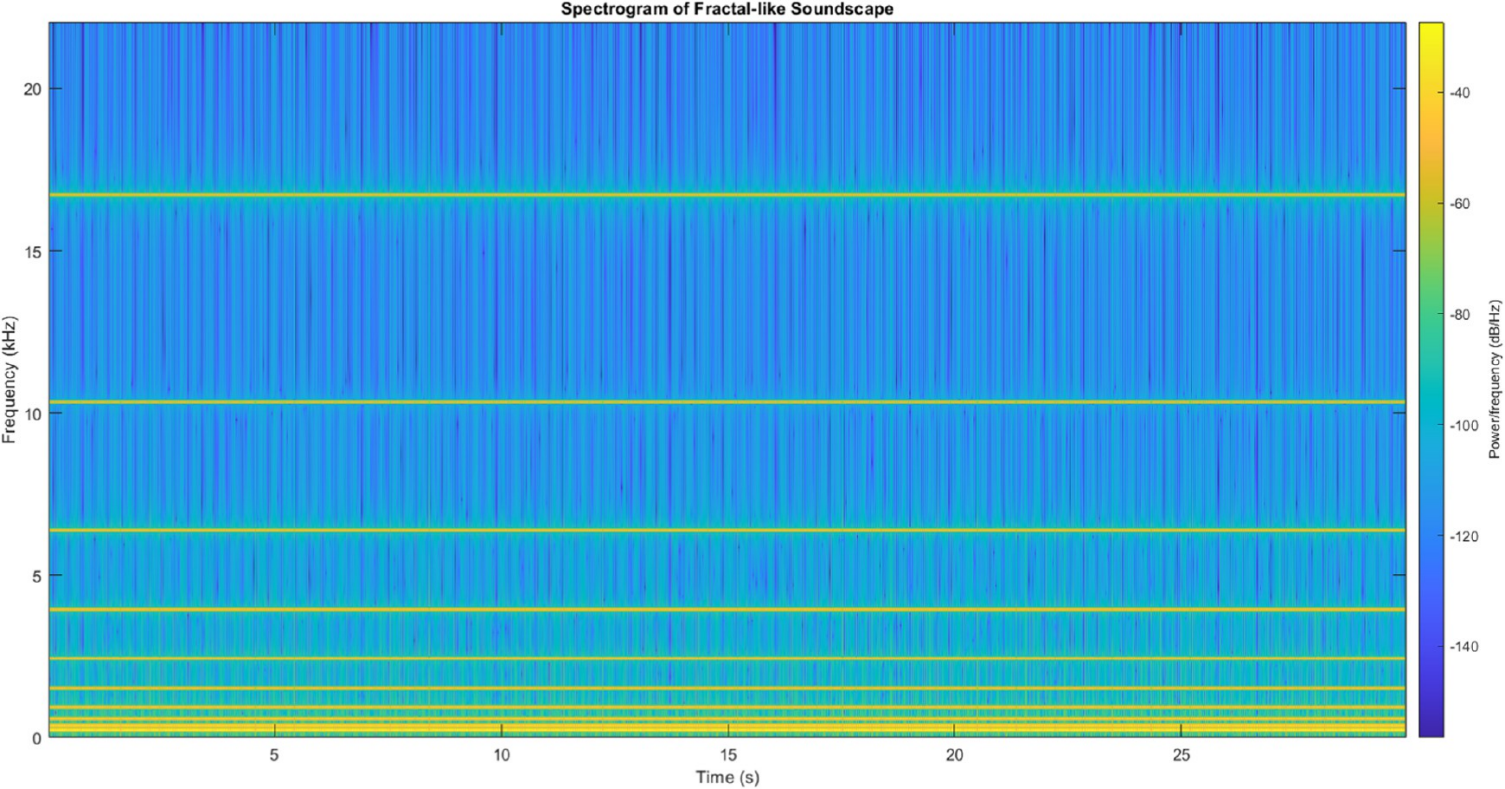
Spectrogram of the fractal-like soundscape.

The subsequent analysis of proteinoid responses to these stimuli will focus on:

1. Frequency response characteristics, *H*(*f*)
2. Temporal integration properties, quantified by the impulse response *h*(*t*)
3. Nonlinear behaviors, assessed through higher-order spectral analysis techniques such as bispectrum *B*(*f*_1_, *f*_2_)

The purpose of these analyses is to clarify any emerging signal processing capacities of the proteinoid systems that may imitate or deviate from conventional biological neural networks.

**Figure 9:**
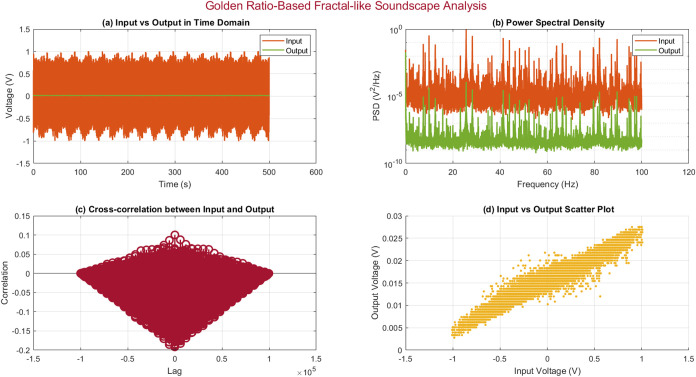
Analysis of the soundscape that is based on the Golden Ratio and exhibits fractal-like characteristics. The time domain plot (a) displays the input and output signals, revealing their complex patterns and similarities. The power spectral density (b) shows clear peaks at specific frequencies, suggesting the existence of harmonics related to the golden ratio in both the input and output signals. The cross-correlation plot (c) shows a significant positive correlation between the input and output, with a maximum correlation coefficient of 0.1916 at a lag of −152 s. (d) Scatter plot demonstrating the clear linear relationship between the input and output voltages, as evidenced by the data points forming a diagonal line.

**Figure 10:**
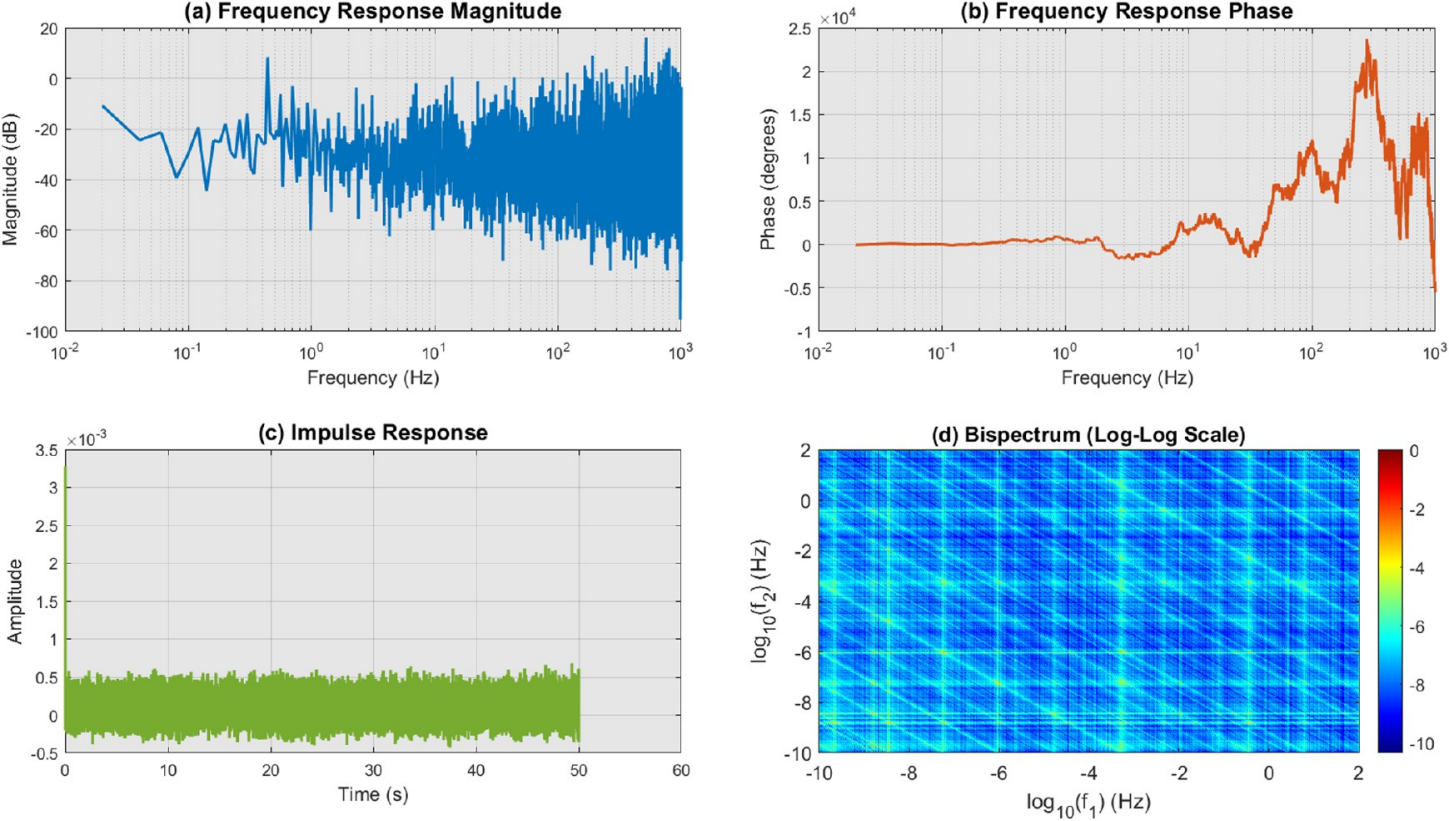
Full examination of the signal processing results obtained from the Golden Ratio experiment. (a) The frequency response magnitude in the top-left panel provides insight into the system’s gain characteristics at various frequencies. (b) The frequency response phase is shown in the top-right panel, which indicates the phase shift caused by the system. (c) The panel in the bottom left demonstrates the impulse response, which describes the time domain behavior of the system. (d) The bispectrum, shown in the bottom-right panel, is a powerful tool for analyzing spectral data. It allows us to capture and understand the nonlinear interactions between different frequency components.

**Figure 11:**
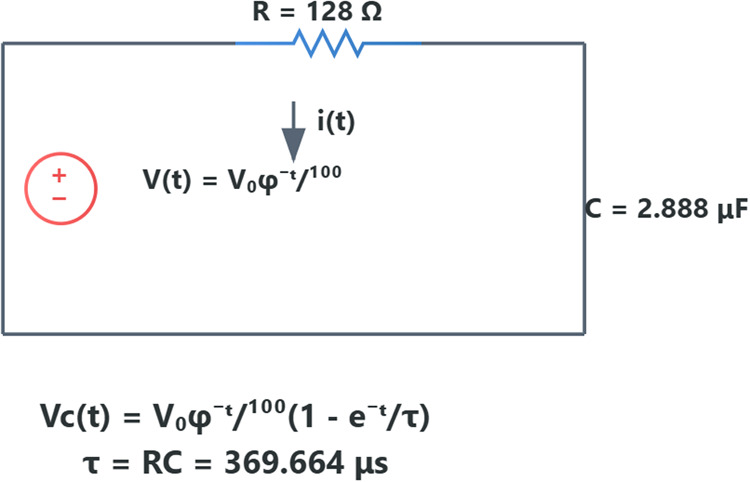
Circuit diagram of the proteinoid system model under Golden Ratio voltage stimulation. The capacitor represents the proteinoid structure, with its capacitance emerging from the arrangement of charged amino acids. The voltage source provides the Golden Ratio-based input, and the resistor models the system’s electrical resistance.

The analysis of the Golden Ratio-based fractal-like soundscape revealed interesting characteristics, as shown in Figures 9 and 10, and summarized in Table 4. Figure 9 presents an in-depth look at the signal properties. The plot in Figure 9a illustrates the complex patterns and parallels between the input and output signals in the time domain. The power spectral density (Figure 9b) shows clear peaks at specific frequencies, indicating the presence of harmonics associated with the golden ratio in both the input and the output signals. The cross-correlation analysis reveals a noteworthy positive correlation between the input and output signals. The maximum correlation coefficient of 0.1916 occurs at a lag of −152 ms (Figure 9c). The scatter plot (Figure 9d) demonstrates a clear linear relationship between the input and output voltages. The analysis yields key metrics, which are presented in Table 4. It is worth mentioning that the input and output signals exhibit distinct voltage characteristics, with average voltages of −0.0190 and 0.0154 V, respectively. The standard deviations for the input (0.2537 V) and output (0.0028 V) signals suggest a notable gap in the signal variability. The correlation coefficient of 0.9649 indicates a robust linear relationship that is evident in the scatter plot. Figure 10 offers a more detailed signal processing analysis. The magnitude of the frequency response (Figure 10a) offers information about the system’s gain at different frequencies, while the phase of the frequency response (Figure 10b) shows the amount of phase shift caused by the system. The impulse response, shown in Figure 10c, describes the time domain behavior of the system. The bispectrum analysis (Figure 10d) provides insights into the nonlinear interactions between frequency components, thereby improving our understanding of the complex dynamics of the system. The *t* test yielded a *p*-value of 0.0000, suggesting a statistically significant distinction between the input and output signals. A sampling frequency of 200.00 Hz is sufficient for capturing the necessary signal characteristics with good resolution. The output audio signal response of proteinoids to this fractal soundscape is provided as the Supporting Information (output_signal.mp3) (Figure 11).

**Table 4:** Key Statistics and Metrics from the Golden Ratio-Based Fractal-like Soundscape Analysis

**parameter**	**value**
input mean voltage	–0.0190 V
input standard deviation	0.2537 V
output mean voltage	0.0154 V
output standard deviation	0.0028 V
correlation coefficient	0.9649
maximum cross-correlation	0.1916
lag at maximum correlation	–152 s
RMS error	0.2533 V
*t* test *p*-value	0.0000
sampling frequency	200.00 Hz

### Capacitive Response of Proteinoid Systems to Golden Ratio Voltage Stimulation

The capacitive behavior of proteinoid systems to voltage stimulation based on the Golden Ratio yields valuable information regarding their potential for use in biocomputing applications. Our experimental setup yielded a capacitance of 2.888 μF, which arises from the inherent properties of the proteinoid structures. The combination of this capacitance and a resistance of 128 Ω results in a time constant τ = RC = 369.664 μs, which describes the dynamics of the system’s response.

Under the applied Golden Ratio voltage stimulation, described by
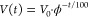
23where *V*_0_ = 10 V and ϕ is the Golden Ratio, the capacitive voltage response of the proteinoid system follows:
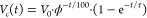
24

This equation represents the relationship between the input based on the Golden Ratio and the capacitive properties of the proteinoid system. The recorded capacitance of 2.888 μF is significant because it indicates the proteinoid structures’ ability to store electric charge. The presence of capacitance in this context is most likely due to the arrangement of electrically charged amino acids within the proteinoid microspheres and their interaction with the surrounding environment. The proteinoid system we observed displays capacitive behavior that is similar to biological membranes. Biological membranes usually have capacitances ranging from 0.5 to 1.0 μF/cm^2^.^63,64^ The increased capacitance seen in our system may suggest a larger surface area or a distinct arrangement of charged molecules within the proteinoid structures, which might improve their ability to process information. The system’s response to the Golden Ratio voltage input demonstrates several critical characteristics:

- Charge accumulation: The proteinoid system’s capacity to accumulate charge rapidly in response to the applied voltage is illustrated by the initial rapid increase in capacitor voltage.
- Nonlinear discharge: The capacitor voltage’s subsequent decay, which mirrors but lags behind the input voltage, demonstrates a nonlinear discharge pattern that is influenced by the system’s RC time constant and the Golden Ratio decay of the input.
- Frequency response: The time constant of 369.664 μs indicates that the system is capable of responding to relatively high-frequency inputs, which could potentially facilitate the processing of information at a rapid pace.

This capacitive behavior, which originates from the proteinoid structures, may function as a fundamental mechanism for the storage and processing of information in our biocomputing system. The system’s capacitive response and the Golden Ratio-based input may result in distinctive computational properties, including frequency-dependent information encoding or nonlinear signal processing. Future research will concentrate on the manner in which this capacitive behavior alters in response to a variety of conditions, including environmental factors or varying proteinoid compositions. Furthermore, the investigation of the interaction between this capacitance and other electrical properties of the proteinoid system has the potential to uncover new paradigms for biomimetic computing, which could lead to the development of innovative information processing architectures that bridge the divide between biological and artificial systems.

Proteinoid computing’s theory comes from their unique biomolecular design. It allows for both signal processing and information storage. The mechanism relies on charged amino acids and their arrangement. They create capacitive networks (*C* = 2.888 μF) with collective electrical behavior. These networks follow nonlinear dynamics described by the Hodgkin-Huxley formalism modified for proteinoid assemblies:
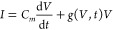
25where *g*(*V*, *t*) represents voltage-dependent conductance arising from amino acid configurations. This theoretical model predicts both the observed frequency-selective response (|*H*(*f*)| ∝ 1/*f*) and nonlinear processing capabilities (THD(*f*) ∝ *f*). Proteinoid systems can compute. Their physical properties mimic biological information processing. The measured capacitance exceeded that of typical biological membranes (0.5–1.0 μF/cm^2^). This suggests better charge storage and processing. The system’s response to Golden Ratio voltage stimulation *V*(*t*) = *V*_0_ϕ^–*t*/100^ shows nonlinear transformation via the capacitive response *V*_*c*_(*t*) = *V*_0_ϕ^–*t*/100^(1 – e^–*t*/τ^), where τ = 369.664 μs enables rapid processing. This behavior, along with frequency-dependent mutual information (*I*(*f*_ϕ_) = 0.142 bits vs *I*(*f*_high_) = 0.023 bits), shows computational functionality. It suggests selective frequency filtering and nonlinear signal transformation. These are like dendritic computation in biological neurons. Dendritic computation in neurons is key to processing info. It integrates and transforms synaptic inputs. This shapes neural responses and the brain’s overall power. Neurons are the nervous system’s basic building blocks. They have a complex, branched dendritic structure. This acts as a “computational unit”. It can perform complex signal processing tasks.^65,66^ The literature describes the diverse forms and functions of neurons. It suggests that each neuronal population has a unique gene expression profile. This profile contributes to their specific roles in the neural network. For instance, central nervous system neurons vary in size and complexity. They differ in the number of dendrites, synaptic connections, axon lengths, and myelination, among other features. This diversity is amplified by including the neurotransmitters’ chemical specificity. They are used for chemical transmission or neuromodulation.^67^ Dendritic computation is key to the neuronal diversity. It allows neurons to process complex information in their dendritic trees. Dendritic integration lets neurons combine and transform multiple synaptic inputs. Dendritic nonlinearities, like voltage-gated ion channels, can boost a neuron’s computing power.^68^

### Fractal-like Soundscapes for Proteinoid Computing

**Figure 12:**
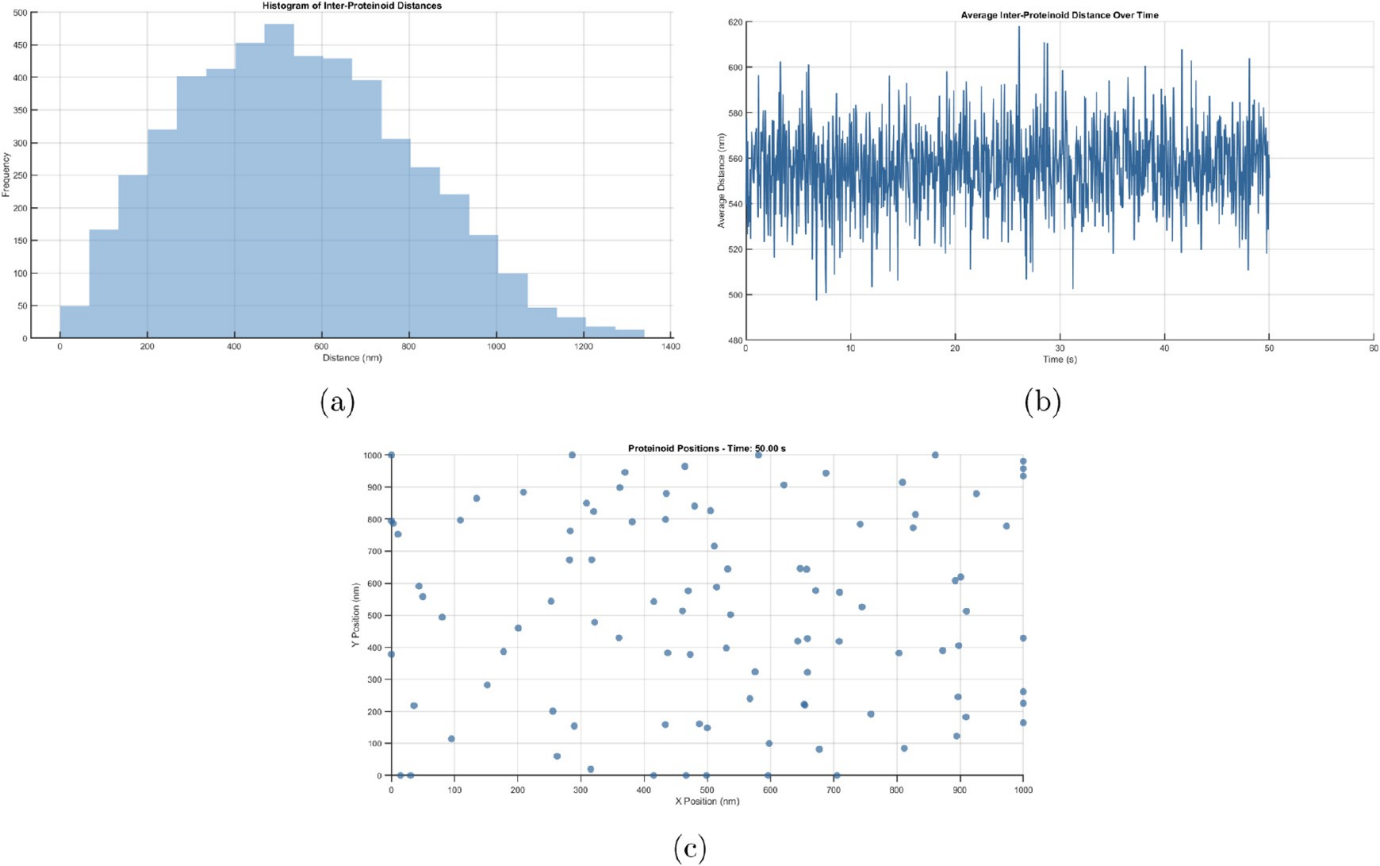
(a) Histogram of inter-proteinoid distances: The movement patterns of proteinoid microspheres are directly influenced by electrical stimulation through electrokinetic effects. When subjected to electrical fields, proteinoids exhibit both electrophoretic motion (due to their surface charge) and dielectrophoretic responses (due to field gradients). These electrical forces contribute to the observed distance distributions. This histogram depicts the distribution of distances across the proteinoids at the end of the simulation. The *x*-axis denotes distance in nanometers (nm), while the *y*-axis illustrates the frequency of occurrence. The distribution seems to be approximately normal with a peak between 600 and 700 nm. This indicates that the majority of proteinoids generally maintain a moderate distance from one another, with limited occurrences of either very near or very distant separations. (b) Average inter-proteinoid distance over time: The high-frequency fluctuations in inter-proteinoid distances correlate with the applied electrical stimulation patterns. The observed oscillations (600–650 nm) are from proteinoids. They respond to the changing electric field. The membrane potential changes, causing movement through conformational adjustments. Proteinoid Positions at 50.00 s: This time series figure illustrates the variations in the average distance between proteinoids during the simulation. The *x*-axis denotes time in seconds, while the *y*-axis indicates the average distance in nanometers. The plot demonstrates fast, high-frequency fluctuations around a mean value of roughly 600–650 nm. The fluctuations indicate that the proteinoids are constantly in motion, affected by the input signal, yet they sustain a rather consistent average spacing over time. [c] This scatter figure illustrates the spatial distribution of proteinoids at the 50 s interval of the simulation. Each point denotes an individual proteinoid, with its location specified by x and y coordinates in nanometers. The proteinoids seem to be uniformly dispersed throughout the 1000 x 1000 nm area, exhibiting no apparent grouping or patterning. The uniform distribution indicates that the simulation conditions facilitate a dispersed organization of proteinoids instead of clustering or division into separate groups.

The proteinoid simulation, as depicted in Figure 12, provides insights into the dynamic behavior of theoretical proteinoid structures under the influence of an external signal. In subfigure [a], we observe the histogram of inter-proteinoid distances at the simulation’s conclusion. The distribution approximates a normal curve, centered around μ ≈ 650 nm, with a standard deviation σ estimated to be about 100 nm. This distribution suggests that the proteinoids maintain a characteristic separation, likely due to a balance between attractive and repulsive forces in the simulated environment. Subfigure [b] illustrates the temporal evolution of the average inter-proteinoid distance *d̅*(*t*). The calculation of this metric at each time step *t* is given by
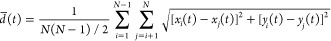
26where *N* is the number of proteinoids and (*x*_*i*_(*t*), *y*_*i*_(*t*)) represents the coordinates of the *i*th proteinoid at time *t*. The rapid oscillations in *d̅*(*t*) reflect the system’s response to the input signal, which modulates the movement of individual proteinoids. The spatial distribution of proteinoids at *t* = 50 s is presented in subfigure [c]. The position update for each proteinoid is governed by the stochastic differential equation:
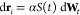
27where **r**_*i*_ is the position vector of the *i*th proteinoid, α is a scaling factor, *S*(*t*) is the normalized input signal at time *t*, and *d***W**_*t*_ represents a Wiener process increment, implemented as a Gaussian random variable in the discrete-time simulation. The physical interpretation of these calculations suggests a system of proteinoids exhibiting Brownian-like motion modulated by an external signal. The consistent average separation implies the presence of effective interaction potentials between proteinoids, which could represent hydrophobic–hydrophilic interactions or electrostatic forces in a real biological system.

The swift variations in average distance indicate that the system is extremely reactive to external stimuli, possibly emulating the behavior of protocells or early biological assemblies in primordial environments. This simulation offers a basic model for examining the collective behavior of proteinoid structures in response to external forces. Although it does not encompass the complete complexities of actual biological systems, it provides significant insights into how fundamental laws might result in emergent behaviors among interacting particles. Further improvements may integrate more realistic contact potentials, diffusion constraints, or chemical reaction kinetics to more accurately reflect the behavior of real proteinoid systems in experimental contexts.

The application of fractal audio soundscapes to affect particle dynamics in our simulation closely parallels research in cymatics, where sound vibrations generate observable patterns in matter. Vuillermet et al.^69^ demonstrated that complex acoustic fields can influence microparticles in three dimensions. Their research demonstrated that particles suspended in fluid media may be arranged into complex shapes using specific acoustic frequencies, similar to the response of our simulated proteinoids to the input signal. This indicates that our model, although simplified, encapsulates fundamental aspects of how acoustic energy can affect the spatial arrangement of microorganisms

Fractal analysis of audio waves has been used to examine complex biological systems. Rodríguez-Liñares et al.^70^ used multifractal analysis to characterize heart rate variability (HRV) signals. Their findings demonstrated that HRV possesses multifractal characteristics, signifying a complex, scale-invariant framework for cardiovascular dynamics. The fractal characteristics of our input signal resemble the complex, multiscale effects found in living systems, paralleling our simulation. This correlation indicates that our model, despite its simplicity, may encapsulate essential elements of living organisms’ responses to complex environmental stimuli. Fractal-based audio analysis has implications in geophysics and environmental monitoring, extending beyond biological systems.

Bianco et al.^71^ used fractal dimension analysis on seismic noise recordings to identify and characterize landslides. Their research revealed that alterations in the fractal characteristics of the surrounding seismic noise could signify slope instabilities. This highlights the potential of employing fractal-like audio signals as a substitute for complex conditions within our simulation. Subsequent iterations of our model may investigate the responses of simulated proteinoids to signals sourced from actual environmental data, potentially providing insights into the interactions between primitive biological structures and their geological environments under early Earth scenarios.

### Analysis of Particle Dynamics and Displacement Patterns in Microfluidic Systems

**Figure 13:**
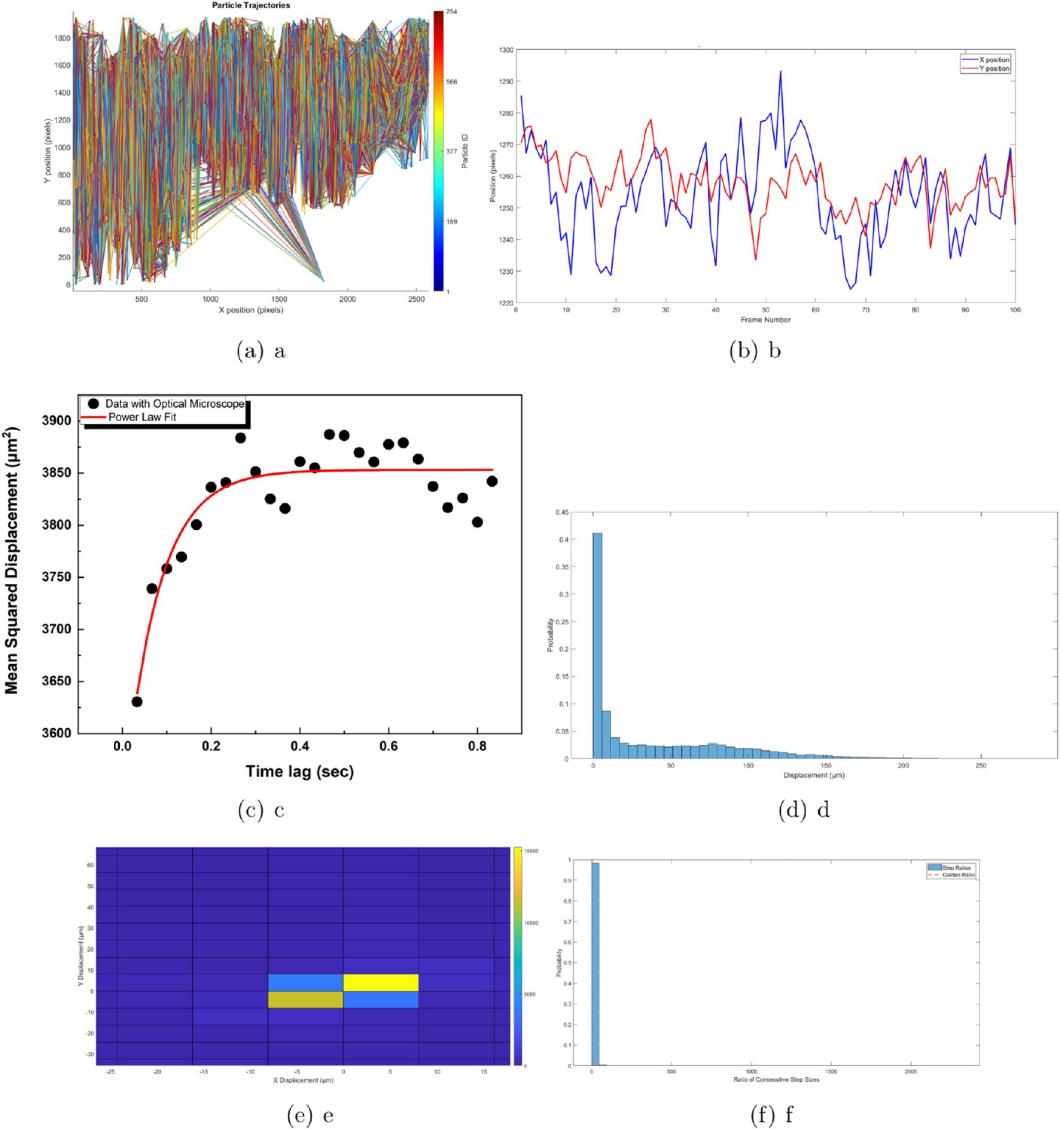
(a) Spatial distribution of particle density. Heatmap showing the concentration of particles across the *X*–*Y* plane, with color intensity indicating particle count. (b) Displacement probability distribution. Histogram depicting the probability of particle displacements highlights the frequency of different movement magnitudes. (c) Mean squared displacement vs time lag. Scatter plot with polynomial fit showing the relationship between mean squared displacement and time lag, indicating diffusive behavior of particles. (d) Temporal evolution of particle positions. Time series plot of *X* and *Y* positions over 100 frames illustrates the dynamic nature of particle movement. (e) Comprehensive particle trajectories. Visualization of multiple particle paths across the observation field, color-coded by particle ID to distinguish individual trajectories. (f) Statistical analysis of particle behavior. Summary of key metrics including mean and median step ratios, diffusion coefficient, and goodness-of-fit measures for polynomial models applied to particle displacement data.

The analysis of particle dynamics was conducted using video microscopy data (see Supporting Video MATV.mp4). Particle trajectories were extracted from the video frames using image processing techniques, including adaptive thresholding and centroid detection. Figure 13a illustrates the trajectories of individual particles over time, providing a visual representation of their complex motion patterns. The position of each particle *i* at time *t* is denoted by **r**_*i*_(*t*) = (*x*_*i*_(*t*), *y*_*i*_(*t*)). The mean displacement of particles over time is presented in Figure 13b, showing the average *x* and *y* positions as functions of the frame number. This is calculated as
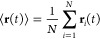
28where *N* is the total number of particles. Figure 13c displays the mean squared displacement (MSD) as a function of the time lag. The MSD was calculated using the equation:
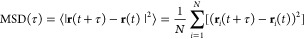
29where τ is the time lag. The mean squared displacement (MSD) data was fitted with an exponential model of the form:
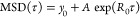
30where τ is the time lag, *y*_0_ is the asymptotic MSD value, *A* is the amplitude, and *R*_0_ is the rate constant. The diffusion coefficient *D* was derived from the linear term of this fit:
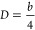
31yielding a value of 1.9908 × 10^2^ μm^2^/s for two-dimensional motion. The distribution of particle displacement magnitudes is presented in Figure 13d, providing insights into the range and frequency of particle movements. The probability density function *P*(Δ*r*) of displacement magnitudes Δ*r* was estimated by using histogram analysis. Figure 13e offers a 2D histogram of displacements, illustrating the spatial distribution of particle motions in the *x*–*y* plane. This represents the joint probability density *P*(Δ*x*, Δ*y*) of displacements in the *x* and *y* directions. An analysis of step size ratios was performed to investigate potential patterns in particle motion. The step size ratio *R*_*i*_ for consecutive steps is defined as
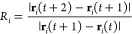
32Figure 13f shows the distribution of these ratios, with the Golden Ratio (ϕ ≈ 1.618) marked for reference. The mean step ratio was found to be 12.1879, with a median of 0.9894 and a standard deviation of 109.0599. The quality of the polynomial fit to the MSD data was assessed using the coefficient of determination (*R*-squared):
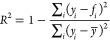
33where *y*_*i*_ are the observed MSD values, *f*_*i*_ are the predicted values from the power-law fit, and *y̅* is the mean of the observed data. The fit resulted in the following parameter values:
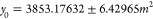
34
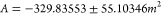
35
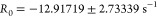
36The quality of the fit is indicated by the reduced χ^2^, the coefficient of determination (*R*^2^), and the adjusted *R*^2^:
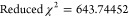
37
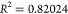
38
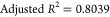
39According to this exponential model, as the time lag rises, the MSD approaches an asymptotic value of about 3853 μm^2^. In accordance with confined or subdiffusive motion, the negative values of *A* and *R*_0_ show that the MSD grows quickly at small time lags before leveling out. Even though there is still some variance that cannot be explained, the model accounts for a sizable amount of the data’s variability, as indicated by the comparatively high *R*^2^ value of 0.82024. The results combined offer an extensive perspective on particle behavior in the observed system, highlighting both diffusive characteristics and perhaps anomalous motion patterns. The elevated diffusion coefficient and extensive range of step ratios signify a highly dynamic system characterized by considerable particle mobility.

**Figure 14:**
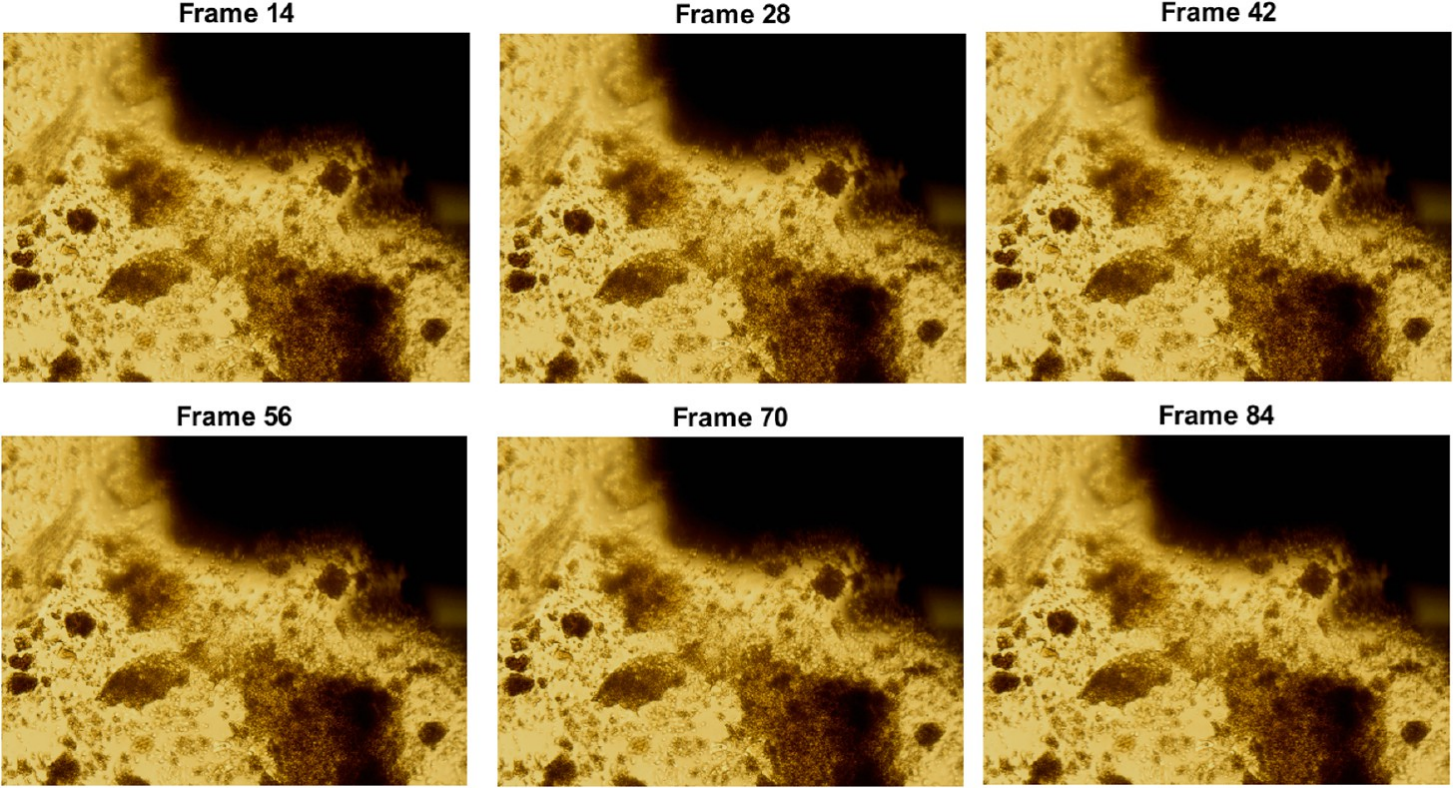
Time-lapse microscopy images of proteinoid microspheres. The spatial distribution and morphology of proteinoid structures over time are demonstrated in six frames (14, 28, 42, 56, 70, and 84) that were extracted from the video recording. The darker regions within the sample denote areas of higher density or depth, while the golden-brown particles represent individual and aggregated proteinoid microspheres. The dynamic behavior of proteinoids, as discussed in the results section, is visually demonstrated by these images.

**Figure 15:**
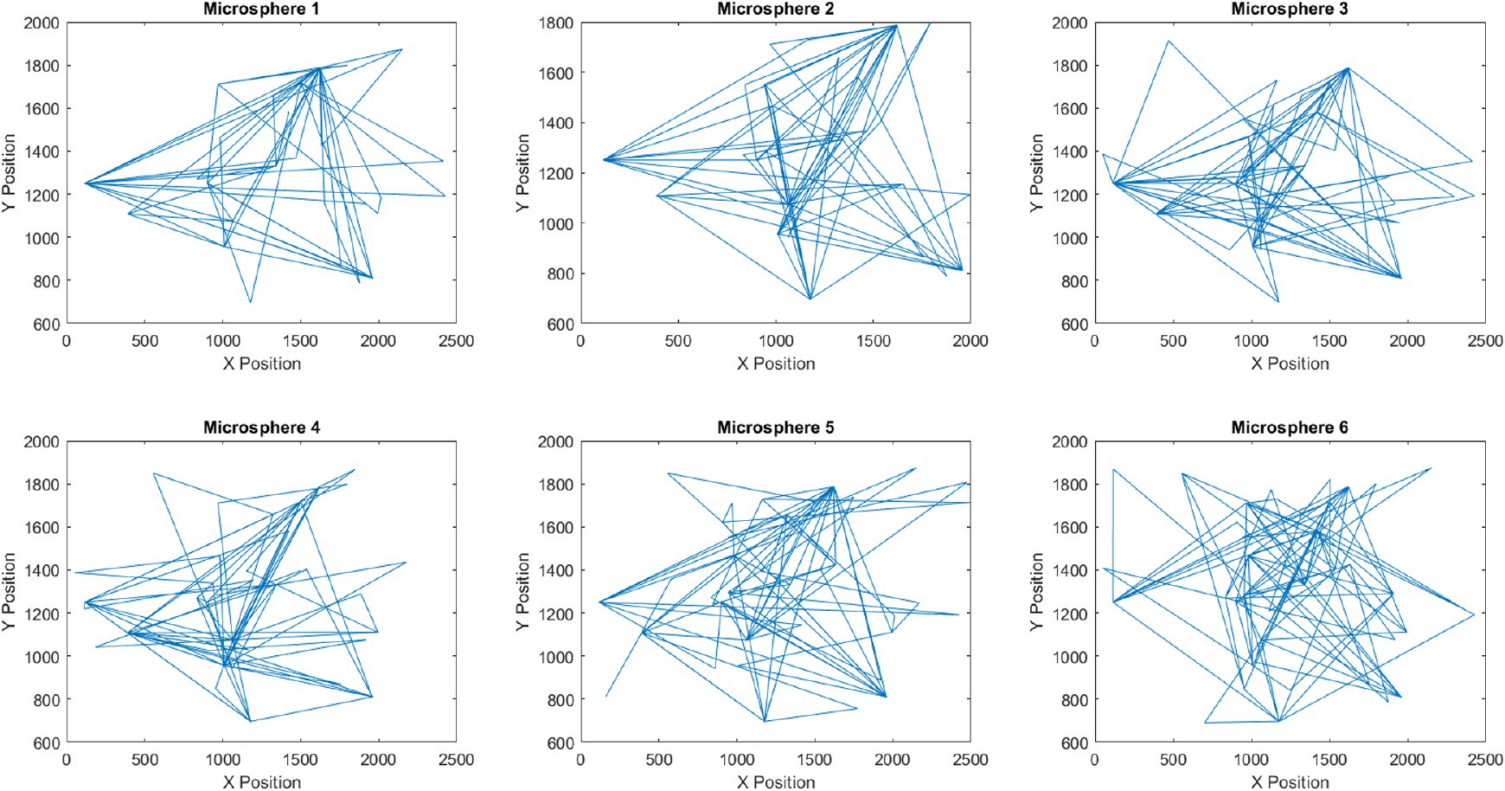
Temporal trajectories of six distinct proteinoid microspheres. Each subplot monitors the two-dimensional trajectory of an individual microsphere, charting its coordinates along the *X* and *Y* axes. The erratic, interrelated trajectories illustrate the complex, dynamic behavior of these minuscule organisms as they interact with their surroundings. The variation in speed and unique paths illustrated in the subplots indicates diversity in the physical characteristics of the microspheres and their reactions to the environment. This visualization clarifies the essential attributes and interconnections of these proteinoid-based microscale systems.

The complex spatial arrangement and subtle movements of proteinoid microspheres over time are revealed in the time-lapse microscopy images, as illustrated in Figure 14. These visual observations provide an expanded view of proteinoid behavior under experimental conditions. The six subplots shown in Figure 15 provide a display of the complex paths that individual proteinoid microspheres take. These microscopic structures are made of molecules based on amino acids that self-assemble, and they behave quite dynamically when they interact with their surroundings.

Each microsphere followed a unique, chaotic route, indicating a significant degree of variation in its physical characteristics and reactions to external stimuli. By examining the statistical distribution of several motion characteristics, such as the mean squared displacement (MSD), which is defined as
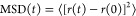
40where *r*(*t*) represents the position vector of a microsphere at time *t*, and the angle brackets ⟨···⟩ denote the ensemble average over all microspheres. Because it provides light on the underlying diffusion mechanisms controlling the mobility of small particles, the MSD is a frequently employed measure in the study of colloidal and biological systems.^72,73^ A more thorough examination of the microsphere trajectories could possibly indicate the existence of anomalous diffusion, which is represented by a power-law expression that describes the nonlinear relationship between the MSD and time:
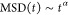
41where the exponent α is indicative of the type of diffusion process, with α = 1 corresponding to normal Brownian motion and α ≠ 1 indicating anomalous diffusion. By fitting the MSD data to this power-law model, one can gain valuable insights into the underlying transport mechanisms governing the motion of proteinoid microspheres. This information, in turn, can shed light on the complex interplay between the physical and chemical properties of these microscale structures and their interactions with the surrounding environment. The rate constant *R*_0_ of −12.90 ± 2.73 s^–1^ was found by the exponential fit of the Mean Squared Displacement (MSD) data. This negative value of *R*_0_ is especially interesting because it implies a rapid initial increase in MSD, which is subsequently followed by a gradual transition to an asymptotic value. The magnitude of |*R*_0_| suggests that this transition occurs on a time scale of approximately 77 ms (1/|*R*_0_|), which is relatively rapid in the context of proteinoid microsphere motion. This rapid approach to an asymptotic MSD value may suggest confined diffusion in which the proteinoid microspheres encounter movement restrictions, potentially as a result of physical barriers or intermolecular interactions within their environment. One may gain valuable insights into the underlying transport mechanisms that govern the motion of the proteinoid microspheres by fitting the MSD data to this power-law model. This information in turn can illuminate the complex connection between the physical and chemical properties of these microscale structures and their interactions with the surrounding environment. Ultimately, the in-depth study of the microsphere trajectories depicted in Figure 15 has the potential to improve understanding of the fundamental principles that underlie the dynamic behavior and self-assembly of these interesting proteinoid-based systems. This has the potential to have a significant impact on a diverse array of applications in fields such as unconventional computing, biotechnology, and materials science.

## Discussion

The investigation of proteinoid systems via Fibonacci sequences and stimuli based on the Golden Ratio provides interesting insights into the integration of synthetic biology and information processing. Although our findings did not provide a direct structural representation of the Golden Ratio in proteinoid microspheres, the system’s reaction to ϕ-based stimuli reveals complex patterns requiring additional exploration. The lack of Golden Ratio proportions in proteinoid structures challenges the sometimes exaggerated universality of ϕ in biological systems. The system’s complex response to ϕ-based stimuli indicates that the importance of these mathematical constants in biology is likely rooted in dynamic processes rather than static structures. This viewpoint aligns with recent research in plant biology, which associates Fibonacci patterns with enhanced energy distribution rather than solely structural aesthetic.^74^ The frequency-dependent response of the proteinoid system to Fibonacci-based tones closely resembles the behavior of biological brain networks. The system’s capacity to analyze and convert complex audio inputs aligns with neuromorphic computing, where researchers strive to replicate brain-like processing in artificial systems.^75^ The proteinoid’s ability to reduce and transform signals may be used in the fabrication of organic computer components, connecting silicon-based technology with biological information processing. The capacitive characteristics demonstrated by the proteinoid system in reaction to Golden Ratio voltage stimulation open new opportunities for bioelectronic interfacing. The measured capacitance, beyond that of standard biological membranes, indicates possible uses in bioinspired energy storage or signal enhancement. This characteristic may be used in the advancement of organic electronic devices, corresponding with the expanding domain of bioelectronics.^76^ The use of fractal-like soundscapes in our research mirrors emerging studies in biophysics and materials science. The approach of employing complex, mathematically based stimuli to affect microscopic structures aligns with current progress in acoustic robotics and matter manipulation.^77^ This methodology may be expanded to regulate the assembly and functionality of synthetic biological systems, providing novel instruments for bottom-up synthetic biology. The nonlinear responses detected in our proteinoid system to ϕ-based stimuli suggest similarities to complex systems theory. The emergent behaviors displayed by these relatively simple structures under particular stimulus environments reflect the principles of self-organization observed in other natural systems, including ecosystems and social networks.^78^ This indicates that proteinoid systems may function as significant models for examining the essential principles of complexity and emergence in both biological and nonbiological contexts. The incorporation of mathematical constants such as ϕ and the Fibonacci sequence into the examination of synthetic biological systems provides novel insights into the significance of mathematics in biology. Although these constants may not be as prevalent in static biological systems as previously believed, their potential significance in dynamic processes and optimal energy distribution requires additional investigation. This methodology corresponds with the expanding domain of mathematical biology, which aims to clarify underlying principles that regulate biological systems using mathematical modeling and analysis.^30^ In conclusion, although our investigation could not validate the structural predominance of the Golden Ratio in proteinoid systems, it revealed complex dynamics in the reaction to ϕ-based stimuli. These findings improve our understanding of proteinoid behavior and open the path for advancements in bioinspired computing, signal processing, and the design of innovative bioelectronic interfaces. Subsequent study ought to concentrate on elucidating the mechanisms underlying these complex reactions and investigating their prospective uses in domains such as biocomputing and materials science.

Our examination of proteinoid ensembles’ responses to Fibonacci sequences uncovers fascinating similarities with other domains where these mathematical patterns are used. The distinctive reactivity and nonlinear amplification effects evident in our proteinoid systems align with the wider applicability of Fibonacci-based methodologies across other fields. The increased sensitivity of proteinoids to particular Fibonacci-derived frequency combinations resembles the efficiency advances observed in the Fibonacci-based encryption schemes. Tarle and Prajapati^1^ showed that these algorithms can rival conventional symmetric key approaches for speed and efficiency. This parallel indicates that the intrinsic characteristics of Fibonacci sequences may provide essential benefits in information processing, applicable to both artificial cryptographic systems and biomimetic structures such as proteinoids. The unique temporal dynamics and emergent oscillatory behaviors found in proteinoid assemblies subjected to voltage patterns inspired by the Golden Ratio reflect the findings of Borysenko et al.^2^ in telecommunications systems. Their research on Fibonacci codes for end-to-end control demonstrated superior error detection capabilities and straightforward architectures, properties that appear to be reflected in our proteinoid systems via improved responsiveness and distinctive pattern generation. Our results align with applications in quantum information. Lai et al.^4^ used Fibonacci compression encoding for quantum secret sharing, resulting in resilient systems with a reduced photon count. Our proteinoid ensembles exhibited improved information processing capacities with Fibonacci-based inputs, indicating a basic efficiency in the interaction of these mathematical patterns with complex systems, whether quantum or biochemical. The extensive applicability of Fibonacci sequences, as illustrated by Tashtoush et al.^5^ in network protocols, reflects the adaptability of our proteinoid reactions. This indicates that Fibonacci-based methodologies may provide universal advantages in system optimization across several domains, ranging from digital networks to biomimetic materials. The parallel with Ketabi and Shahtahmasebi’s^6^ work on electronic systems is particularly noteworthy, as Fibonacci chains demonstrated nonlocalized states and transparent states around the Fermi level. Our observations of distinctive structural configurations and computational abilities in Fibonacci-inspired proteinoid systems may reflect analogous underlying principles, indicating fundamental relationships between these mathematical sequences and the behavior of complex, self-organizing systems. In summary, our research on proteinoid ensembles improves our understanding of these biomimetic systems and adds to the accumulating evidence that Fibonacci sequences and associated mathematical principles may provide universal benefits in information processing, structural organization, and system optimization across various domains. These discoveries create new opportunities for research into the essential function of mathematical patterns in biological and biomimetic systems with possible applications extending from advanced materials to information security and beyond.

**Figure 16:**
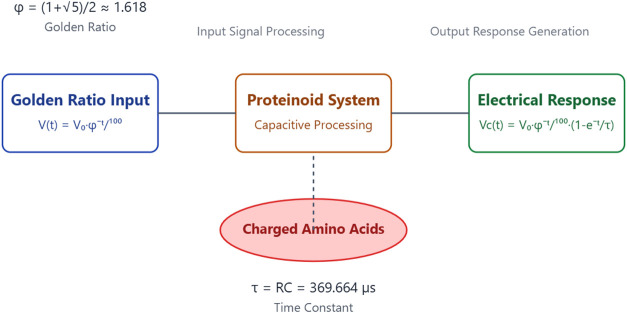
Conceptual mechanism of proteinoid electrical response to stimulation by the Golden Ratio. The diagram depicts the progression of signal processing from input to output via the proteinoid system. The Golden Ratio (ϕ ≈ 1.618) underlies the input voltage decay curve *V*(*t*). The power spectral density (PSD) charts for the input and output signals illustrate the system’s frequency-dependent processing capabilities. The proteinoid structure, composed of charged amino acids, exhibits a capacitive effect defined by time constant τ. This capacitance affects the conversion of the input signal, yielding the output voltage *V_c_*(*t*). The equation for *V_c_*(*t*) integrates both the decay of the Golden Ratio and the exponential charging characteristics inherent in capacitive systems. This model indicates a complex relationship between the mathematical characteristics of the Golden Ratio and the biophysical features of proteinoid structures, potentially resulting in distinctive signal processing abilities that connect synthetic and biological computing paradigms.

Figure 16 depicts the mechanism of proteinoid electrical reaction to Golden Ratio stimulation, offering a description of the system’s behavior. The input signal, described by the equation *V*(*t*) = *V*_0_·ϕ^–*t*/100^, incorporates a voltage drop based on the Golden Ratio into the proteinoid system. This input uses the mathematical characteristics of ϕ (about 1.618), perhaps eliciting resonant behaviors in the proteinoid structures. The power spectral density graphs for both input and output signals indicate differences in frequency content, implying complex signal processing within the proteinoid system. The input PSD exhibits a wider frequency range, whereas the output PSD demonstrates a more filtered response, notably attenuating higher frequencies. The filtering effect may be attributed to the capacitive characteristics of the proteinoid system, resulting from the configuration of charged amino acids. The capacitive effect, denoted by the time constant τ = RC = 369.664 μs, is essential in determining the electrical response. The short time constant indicates that the proteinoid system can swiftly react to input variations, facilitating high-frequency information processing. The output voltage equation, *V*_*c*_(*t*) = *V*_0_·ϕ^–*t*/100^·(1 – e^–*t*/τ^), integrates the effects of the Golden Ratio input and the system’s capacitive characteristics. This mechanism highlights the potential of proteinoid systems as bioinspired signal processors, capable of converting inputs in ways that may emulate specific features of organic brain networks. The incorporation of Golden-ratio-based stimulation with the inherent characteristics of proteinoids creates opportunities for investigating biomimetic computing models and unconventional information processing architectures.

The Landauer principle explains the thermodynamics of information processing in biology.^41^ It defines the minimum energy loss for irreversible logical operations. Erasing one bit of information requires a minimum energy dissipation of *kT* ln 2. Here, *k* is the Boltzmann constant, and *T* is the absolute temperature.^42^ In the context of proteinoid ensembles, the prevalence of Fibonacci patterns may represent an evolutionary adaptation to minimize the energetic cost associated with biological information processing.^79^ Theoretically, copying information can happen without losing it. But, erasing or changing it has unavoidable thermodynamic costs. The responsiveness of proteinoid ensembles to Fibonacci sequences suggests a link. These patterns may be nature’s way to optimize information processing in biology. This optimization fits with a broader idea. Biological structures evolved to save energy while keeping their complexity.^80^ Fibonacci sequences are ordered and predictable. They reduce the computational complexity of biological systems. This minimizes the energy costs of processing and storing information, per the Landauer principle.^81^

## Conclusions

Our research links ancient mathematical concepts with modern biocomputing. It offers a new way to build cognitive systems. These systems would better echo nature’s fundamental patterns. We systematically studied proteinoid responses to Fibonacci sequences- and Golden Ratio-based stimuli. Our work has revealed several significant findings. We tested multiple signal types: linear voltage ramps (0.1–10 V), sinusoidal waveforms (1–100 Hz), and random noise patterns. The proteinoid systems reacted uniquely to sounds based on the Fibonacci sequence. They were more sensitive to certain frequency combinations and nonlinear amplification effects. This suggests potential applications in bioinspired signal processing and information encoding. Our analysis found that proteinoid assemblies have complex, time-varying behaviors. They oscillate when exposed to Golden-ratio-based voltage patterns. These behaviors were not seen with conventional input signals. This suggests that mathematically structured stimuli may be important in biological information processing. The proteinoid systems have a capacitance of *C* = 2.888 μF and a response time of τ = 369.664 μs. These traits may enable high-frequency data processing. They could lead to new methods for biomimetic computing. We found no direct signs of the Golden Ratio in proteinoid organization. However, the system’s complex response to ϕ-based stimuli suggests that mathematical principles may play a subtler role in dynamic biology than in static structures. These findings open new avenues for research in several key directions. We envision bioinspired computing architectures. They would use natural mathematical patterns. We also want to design new bioelectronic interfaces based on proteinoid systems. Our work allows a deeper look at the principles of information processing in early chemical systems. It also supports the creation of new biomimetic materials with better information processing. The natural world’s complex designs inspire a convergence of biology, math, and computing. This could create systems that are more efficient, adaptable, and robust. Our work suggests that using math, like the Fibonacci sequence and Golden Ratio (ϕ), in synthetic biology may spark innovations. They could impact fields from unconventional computing to biomedicine.

## Data Availability

Data is available on the Zenodo database at: https://zenodo.org/records/13858434.

## Supporting Information Available

Analysis of particle dynamics (MP4)
OMEGA (ZIP)
EIS characterization of proteinoid l-Glu:l-Phe:l-Asp (Figure S1); proteinoid synthesis apparatus (Figure S2); detailed schematic of the experimental setup designed for analyzing proteinoid responses to Fibonacci Fractal-like Soundscape stimuli (Figure S3); experimental configuration for proteinoid-kombucha bioelectrical measurements (Figure S4); waveform representation of Combined Fibonacci Tones (Figure S5); and waveform of the Fractal-like Soundscape (Figure S6) (PDF)
