# Correction to “On the Response of Proteinoid Ensembles to Fibonacci Sequences’’

**DOI:** 10.1021/acsomega.5c03031

**Published:** 2025-05-07

**Authors:** Panagiotis Mougkogiannis, Andrew Adamatzky

We are issuing an addition to the acknowledgment section of the original manuscript. The corrected acknowledgment is below. We apologize for the oversight.

