# Supplementary material for: On the Response of Proteinoid Ensembles to Fibonacci Sequences
Source: ACS Omega. 2025 Mar 5;10(10):10401–24. doi: 10.1021/acsomega.4c10571 (PMC11923683; doi:10.1021/acsomega.4c10571)
Supplement: Supplementary file 3 — ao4c10571_si_003.pdf [file ao4c10571_si_003.pdf]

Supporting Information

On response of proteinoids ensembles to Fibonacci sequences

Panagiotis Mougkogiannis[1,*] and Andrew Adamatzky[1]

[1]Unconventional Computing Laboratory, University of the West of England, Bristol, UK

Email: Panagiotis.Mougkogiannis@uwe.ac.uk

EIS analysis of proteinoid L-Glu:L-Phe:L-Asp (Figure S1) showed: (a) a Log-Log plot of impedance magnitude —Z"— vs —Z—. It had a semicircle, indicating a parallel RC circuit, with a minimum at about $2.2 \times 10^4\ \Omega$. This suggests charge transfer at the proteinoid-electrode interface; (b) a Bode plot of impedance magnitude —Z— (blue) and phase angle (red). It revealed a decrease in impedance with increasing frequency and phase behavior typical of the proteinoid's electrochemical response; and (c) a plot of capacitance vs frequency. It showed frequency dispersion, with capacitance dropping from $\sim 10^{-5}$ F at low frequencies to $\sim 10^{-10}$ F at high frequencies (10^5 Hz). This indicates distributed time constants due to the heterogeneous nature of the L-Glu:L-Phe:L-Asp proteinoid structure and its interface with the electrolyte. The EIS data suggests a complex charge transfer mechanism within the amino acid-based proteinoid system.

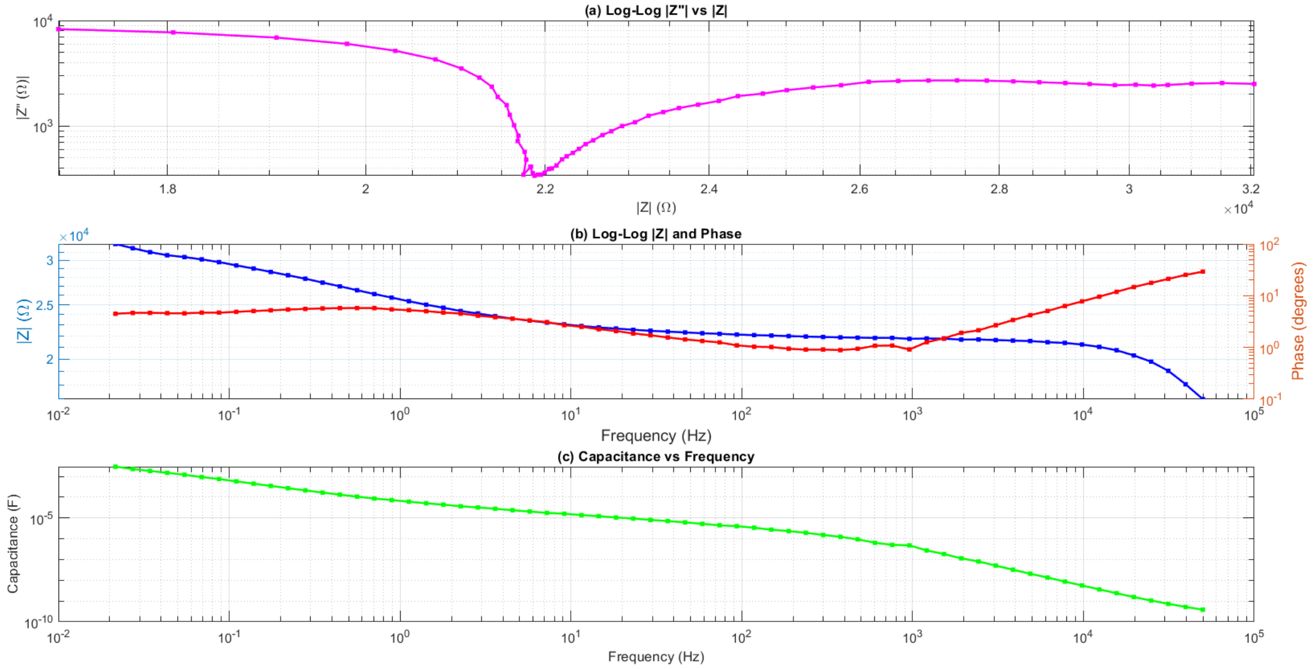

Figure S1: Impedance spectroscopy analysis showing: (a) A Log-Log plot of —Z"— vs —Z—. It displays a semicircle, indicating a parallel RC circuit, with a minimum at about $2.2 \times 10^4\ \Omega$. (b) A Bode plot of —Z— (blue) and phase angle (red). It shows a decrease in impedance with increasing frequency and phase behaviour of electrochemical interfaces. (c) A capacitance vs frequency plot. It shows capacitance decreasing from $\sim 10^{-5}$ F at low frequencies to $\sim 10^{-10}$ F at high frequencies (10^5 Hz). This suggests distributed time constants in the electrochemical system. The data indicates a complex electrode-electrolyte interface with multiple relaxation processes.

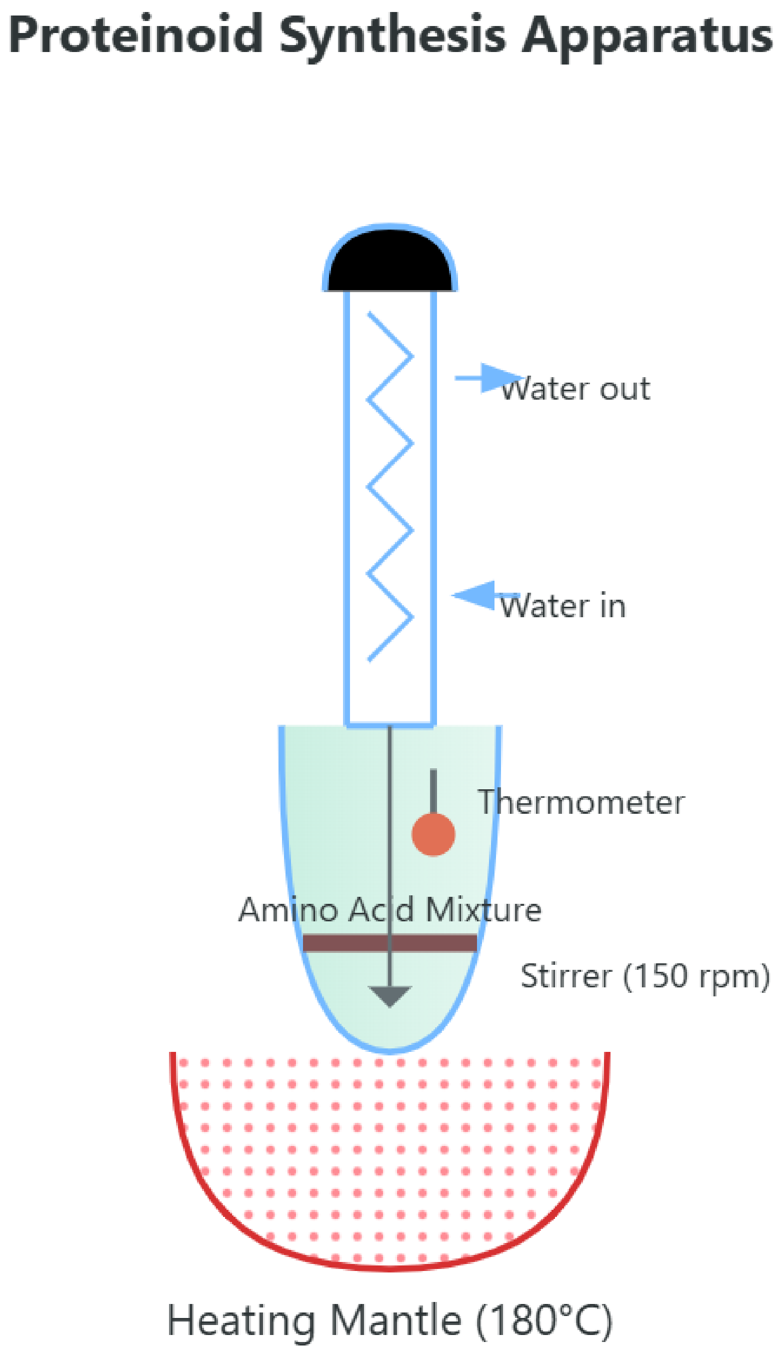

Figure S2: A schematic of the proteinoid synthesis apparatus. It shows a 50 mL round-bottom flask with a reflux condenser.The flask is heated using a heating mantle set to 180°C, with stirring at 150 rpm, and monitored by a thermometer. Amino acid mixture (L-glutamic acid, L-phenylalanine, L-aspartic acid) is heated to form brownish melt. Water inlet/outlet for condenser cooling system indicated by blue arrows.

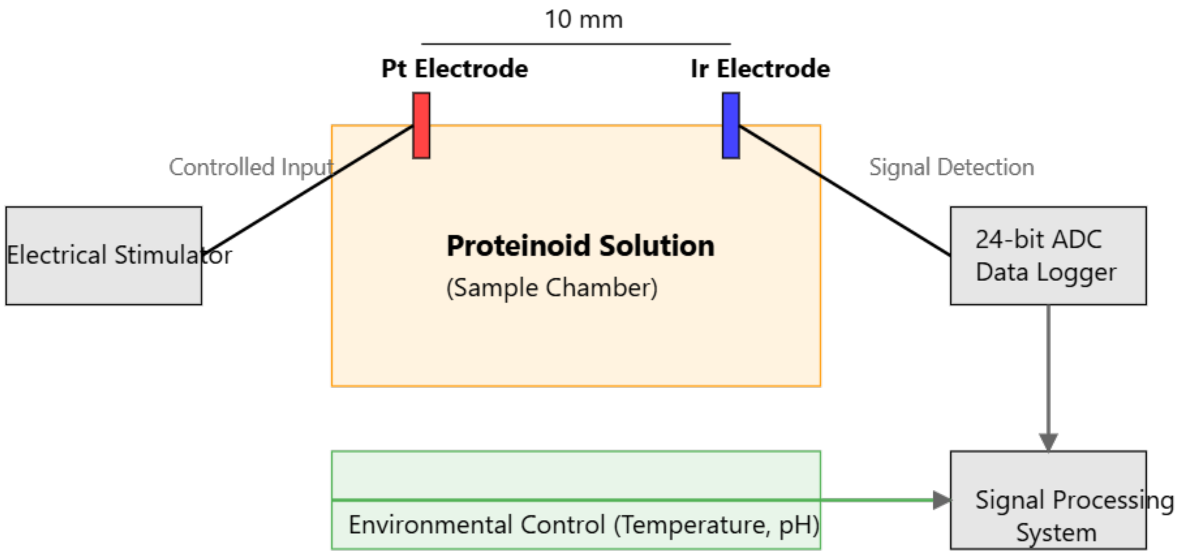

Figure S3: Experimental setup for analyzing proteinoid responses to Fibonacci Fractal-like Soundscape stimuli. Data processing pipeline consists of: (1) Audio signal digitization at $f_s = 44.1$ kHz with 16-bit resolution, (2) MATLAB-based signal preprocessing using a Butterworth bandpass filter ($f_{\text{low}} = 0.1$ Hz, $f_{\text{high}} = 1$ kHz, order $n = 4$), (3) Generation of Fibonacci-based frequencies $f_n = f_0 \phi^n$ Hz, where $f_0 = 220$ Hz and $n = 0, 1, ..., 9$. The BK Function Generator delivers voltage waveforms ($V_{\text{pp}} = 10$ V) through platinum and iridium electrodes (diameter $d = 0.2$ mm, separation $l = 10$ mm) to the proteinoid solution chamber ($V = 50$ mL). Environmental parameters (temperature $T = 25 \pm 0.1°$C, pH $= 7.0 \pm 0.01$) are maintained by a feedback control system. Response signals are acquired using a PicoScope (100 MHz bandwidth, 1 GSa/s) with 24-bit vertical resolution ($\Delta V = 0.97$ μV). Power spectral density estimation employs Welch's method ($N_{\text{FFT}} = 4096$, Hanning window, 50% overlap).

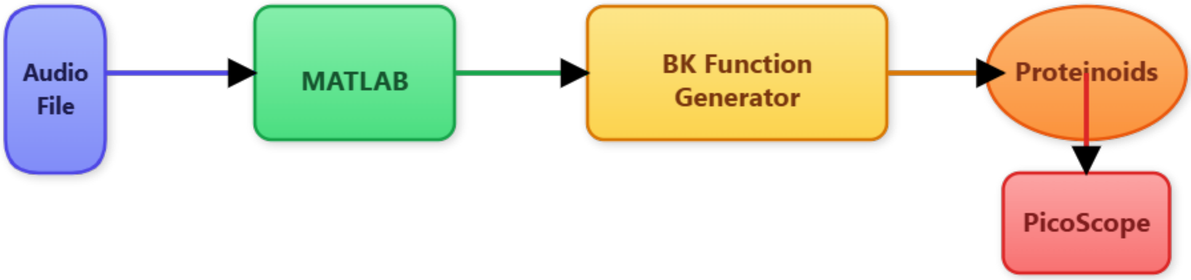

Figure S4: Experimental setup for analyzing proteinoid responses to Fibonacci Fractal-like Soundscape stimuli. The central chamber has a proteinoid-kombucha solution. Platinum and iridium electrodes (0.2 mm diameter) monitor it. They are fixed 10 mm apart. We used a PicoScope 4000 oscilloscope (2 Channel 100 MHz-1 GSa/s) to acquire signals. It has a 24-bit ADC for high-precision, microvolt-range voltage measurements (resolution: 0.97 μV). Environmental parameters were continuously monitored using a PicoLog ADC-24 system (temperature: $\pm 0.1°$C, pH: ± 0.01). The BK Precision 4053 function generator delivered Fibonacci-based stimulation patterns (freq: 0.1 Hz to 1 kHz, amp: 0-10 V peak-to-peak) through iridium-coated stainless steel electrodes. We processed raw data using custom MATLAB scripts. They used Welch's method to estimate the power spectral density. We applied a 4096-point FFT, a Hanning window, and 50% overlap. Signal filtering employed a combination of Butterworth bandpass (0.1-100 Hz) and notch (50/60 Hz) filters to remove environmental noise. Cross-correlation analysis and statistical processing were performed using MATLAB's Signal Processing Toolbox (v2023b). The complete data acquisition and processing pipeline maintained timing precision through hardware-triggered synchronization between stimulation and recording systems.

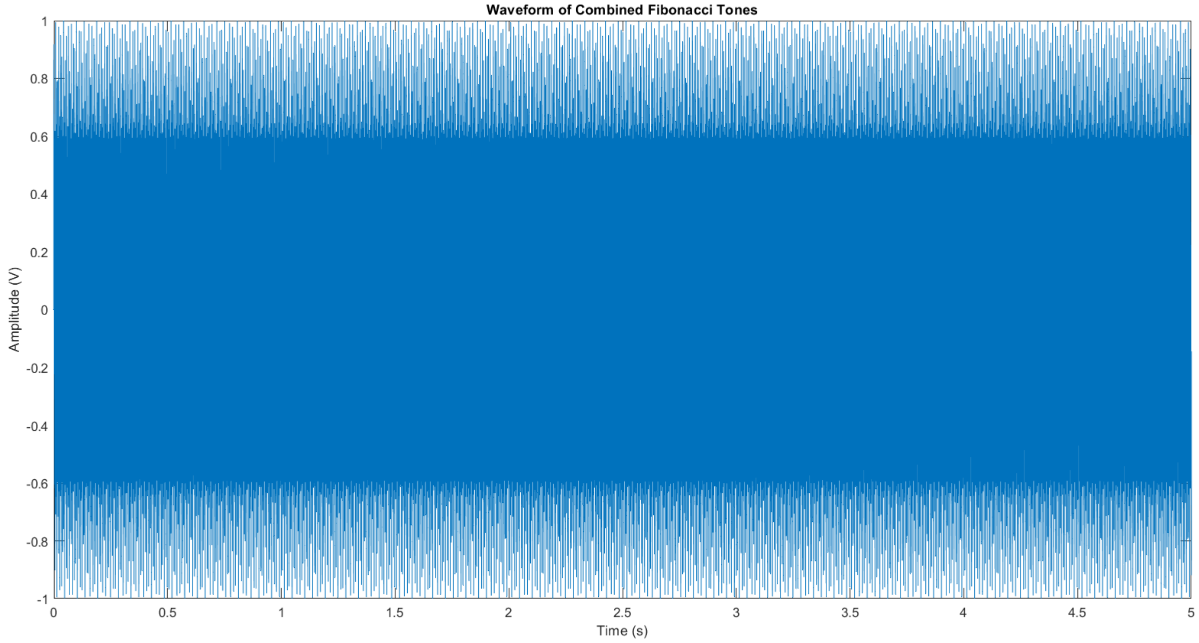

Figure S5: Waveform of Combined Fibonacci Tones

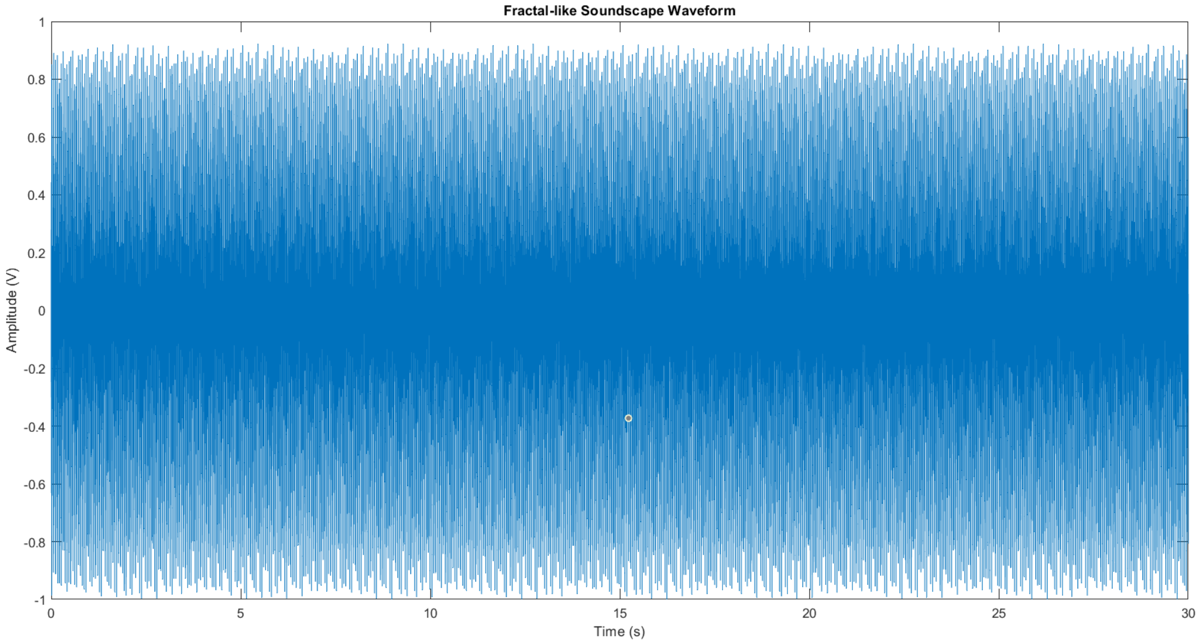

Figure S6: Waveform of Fractal-like Soundscape.
